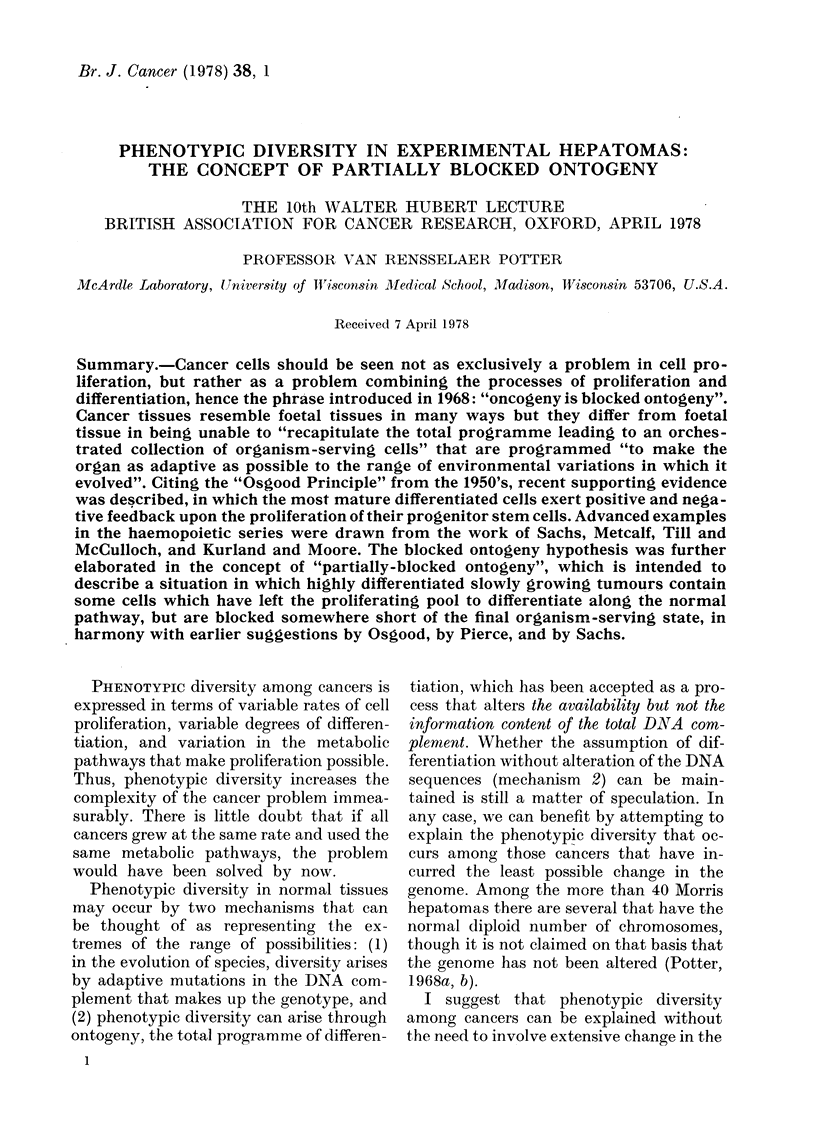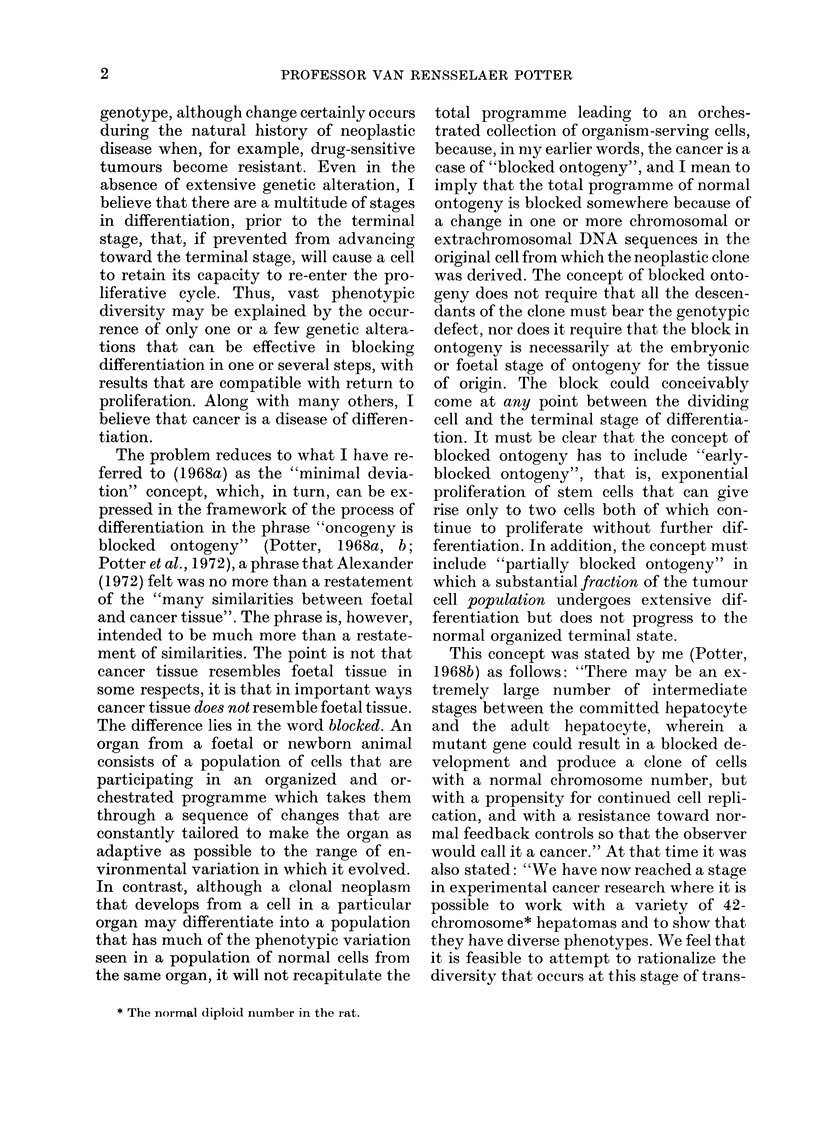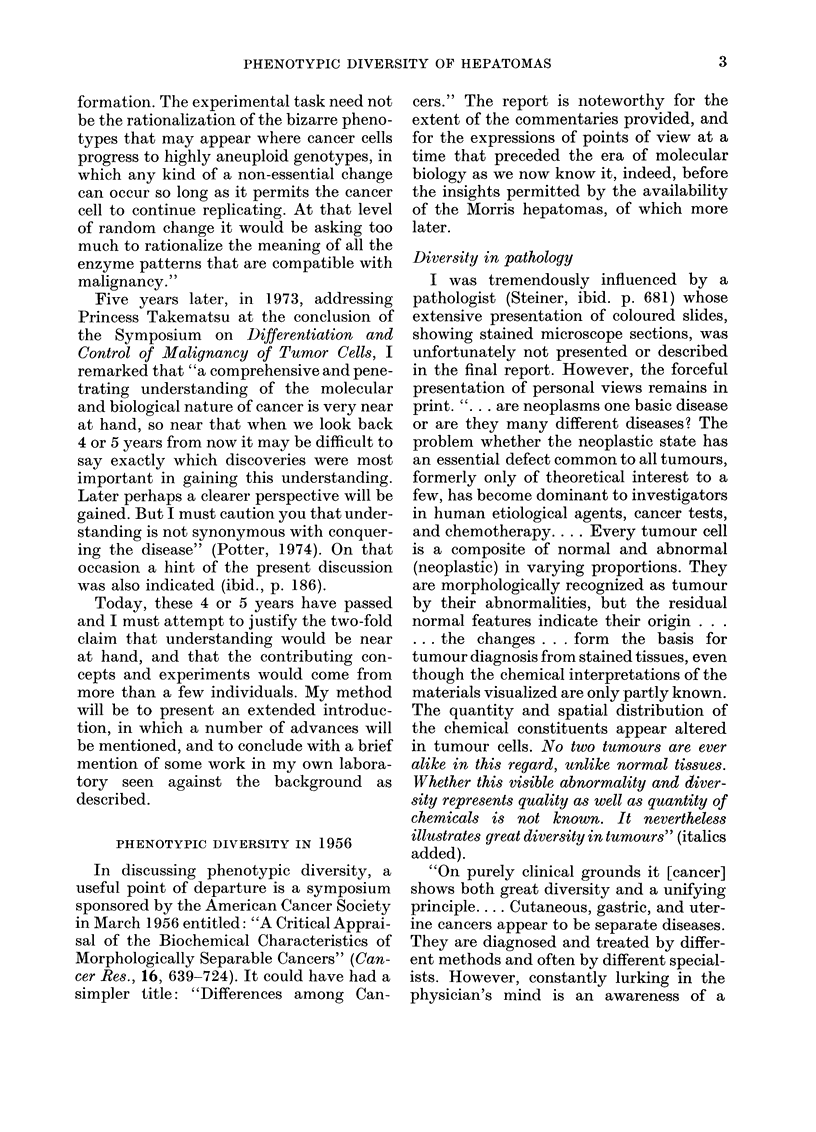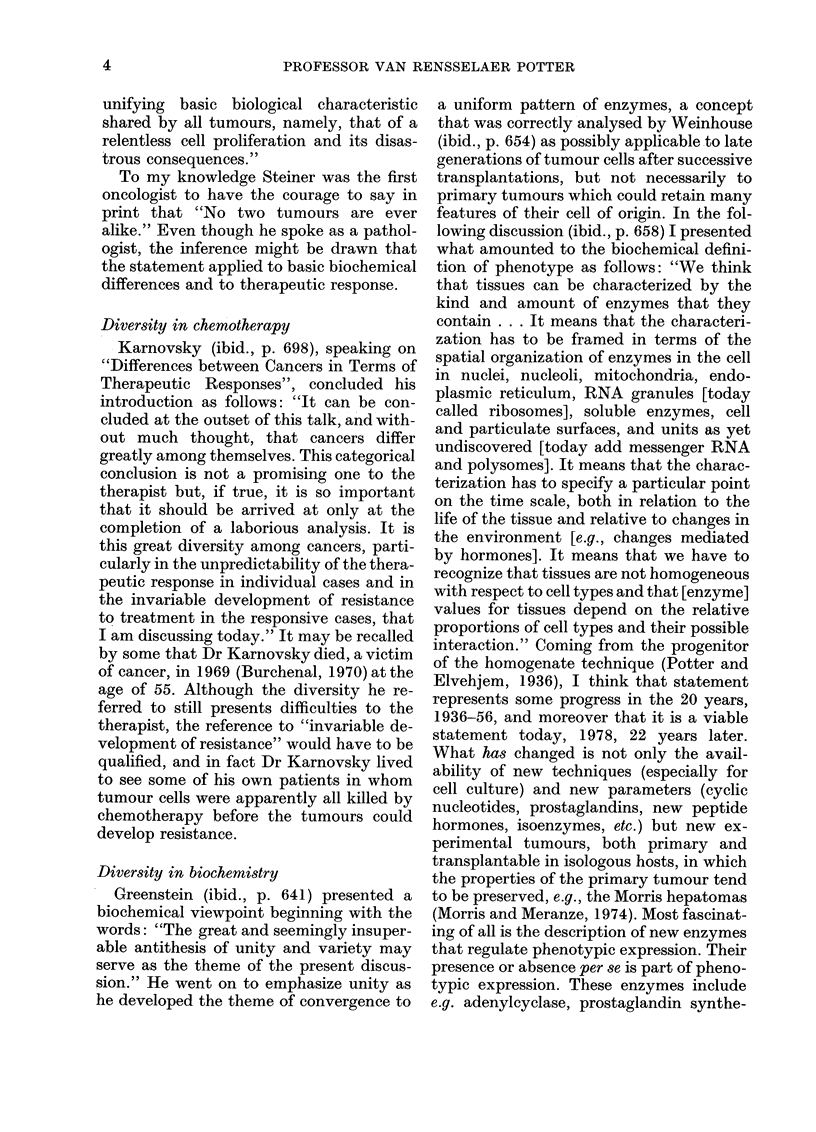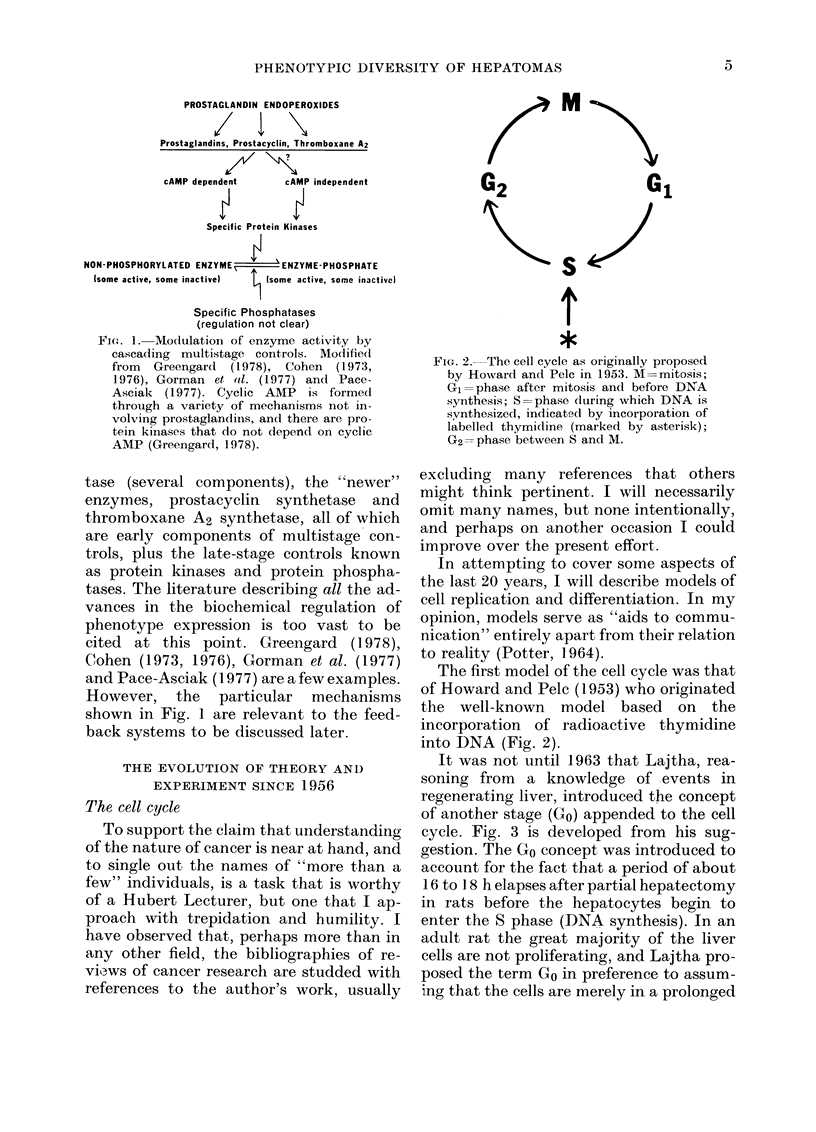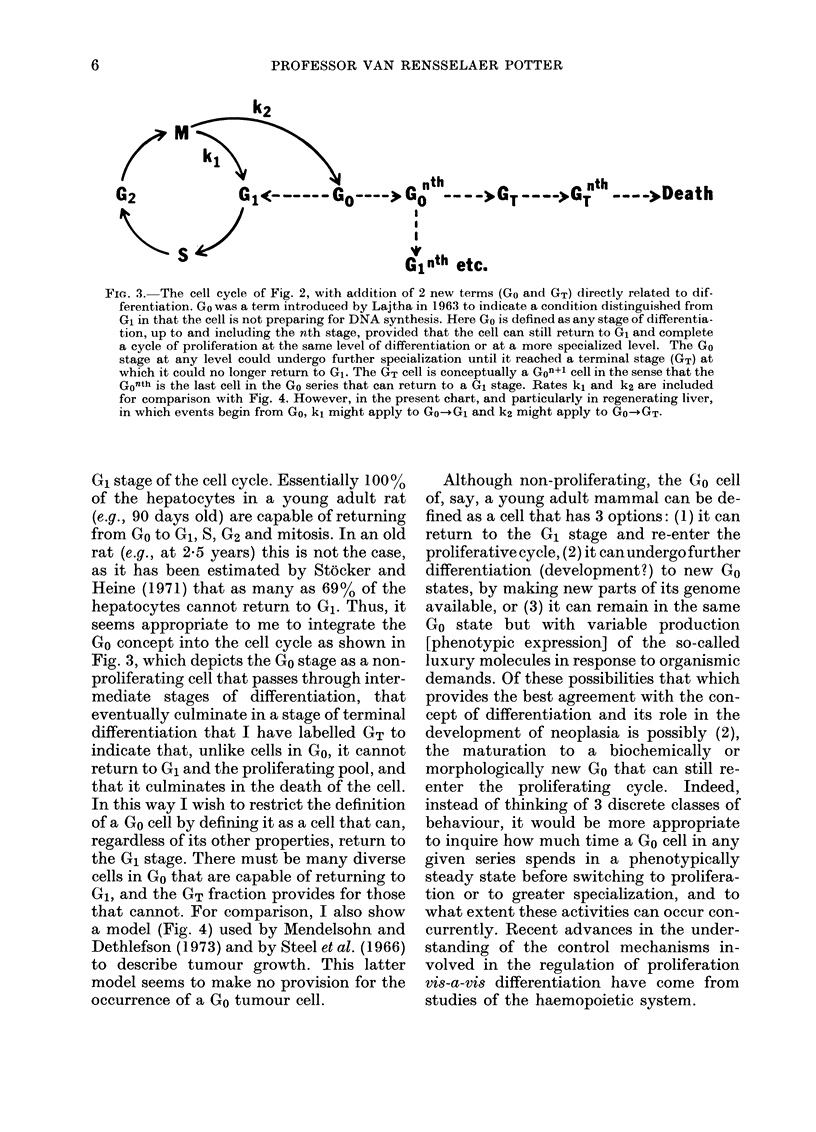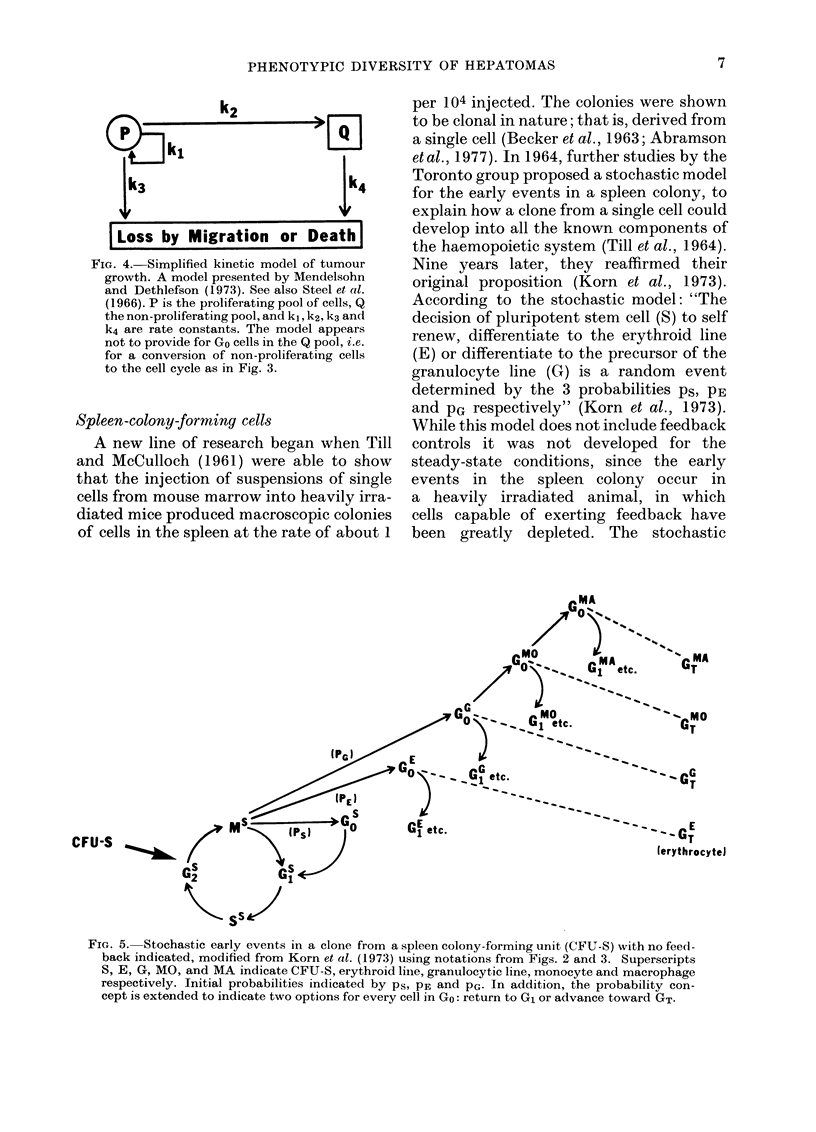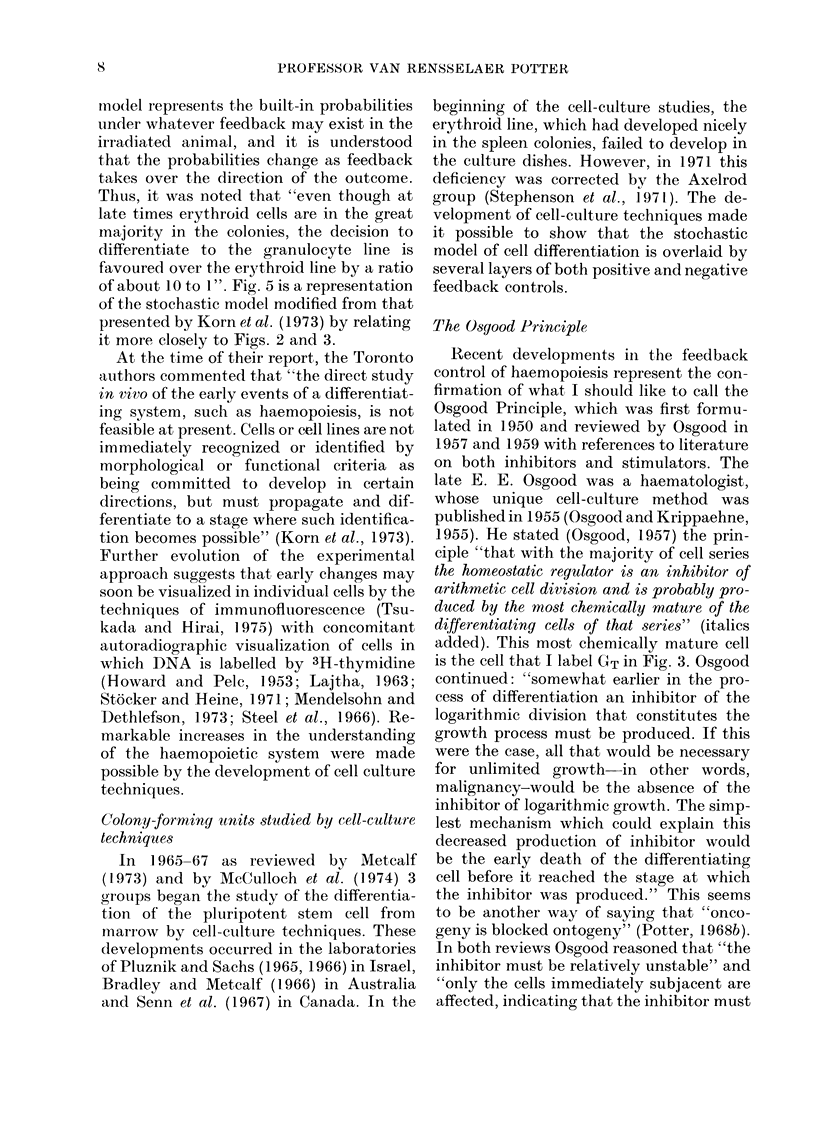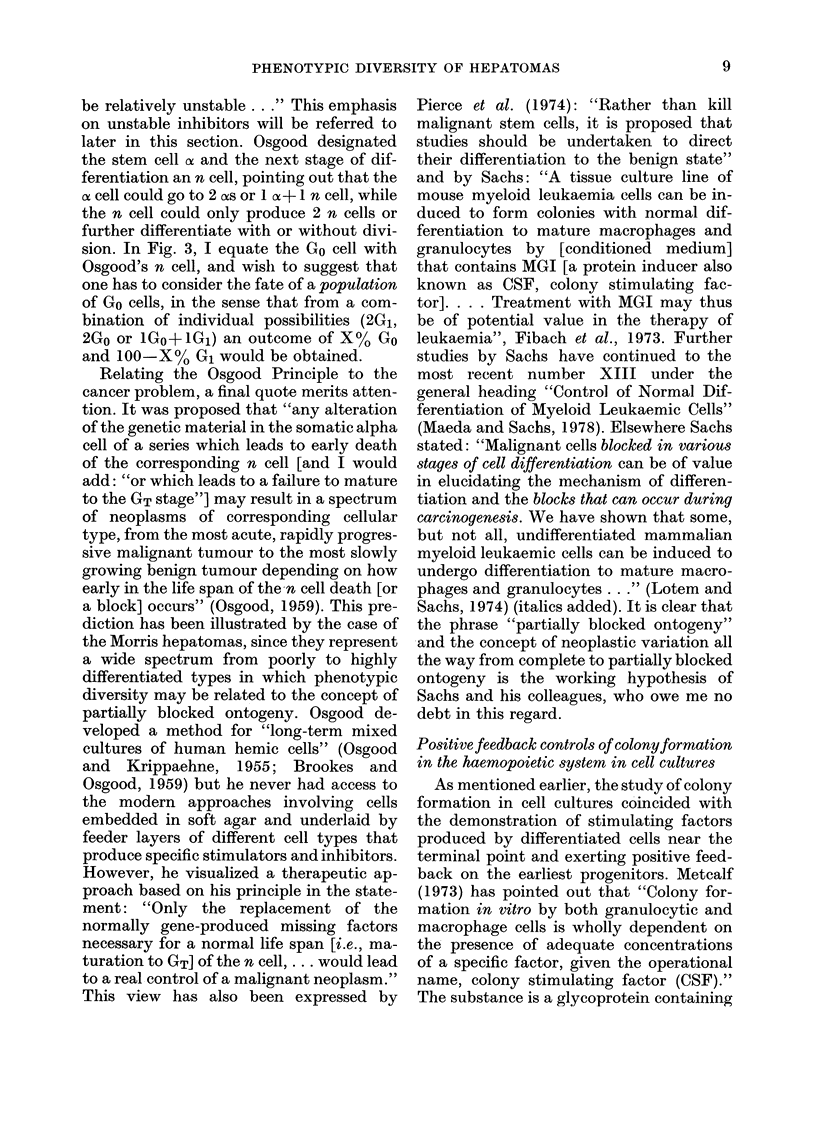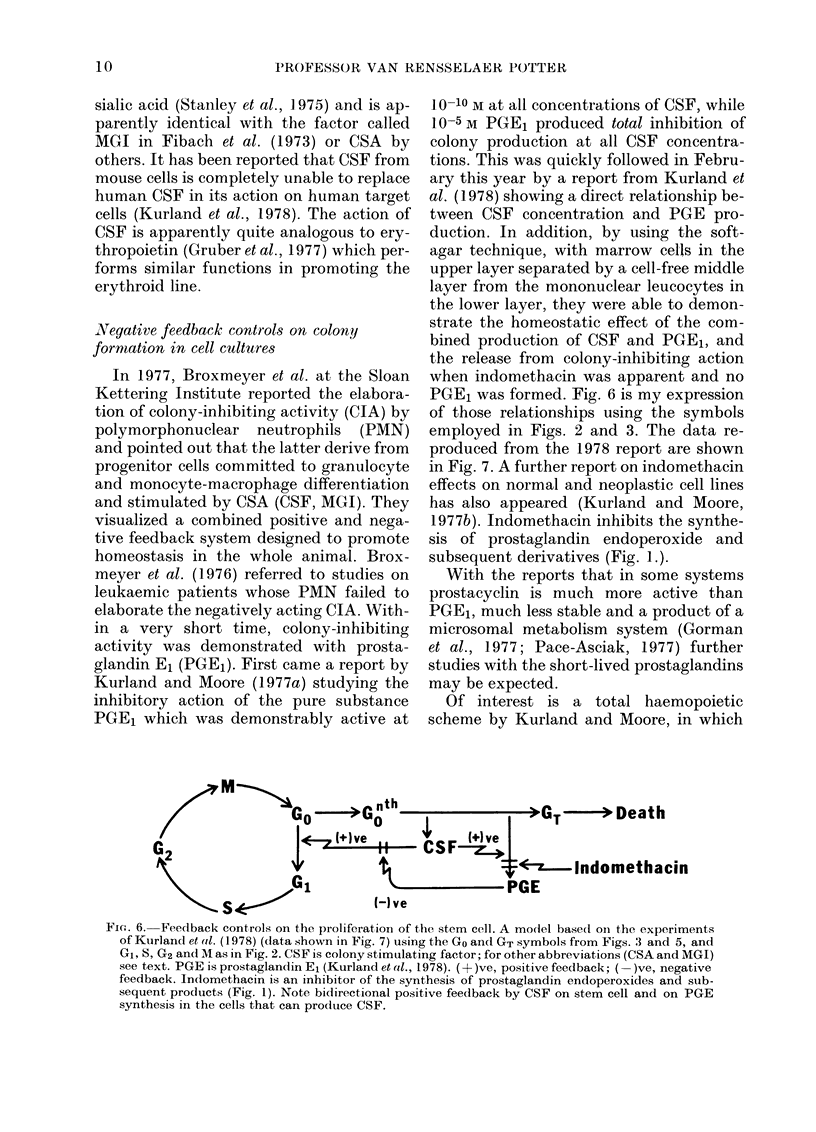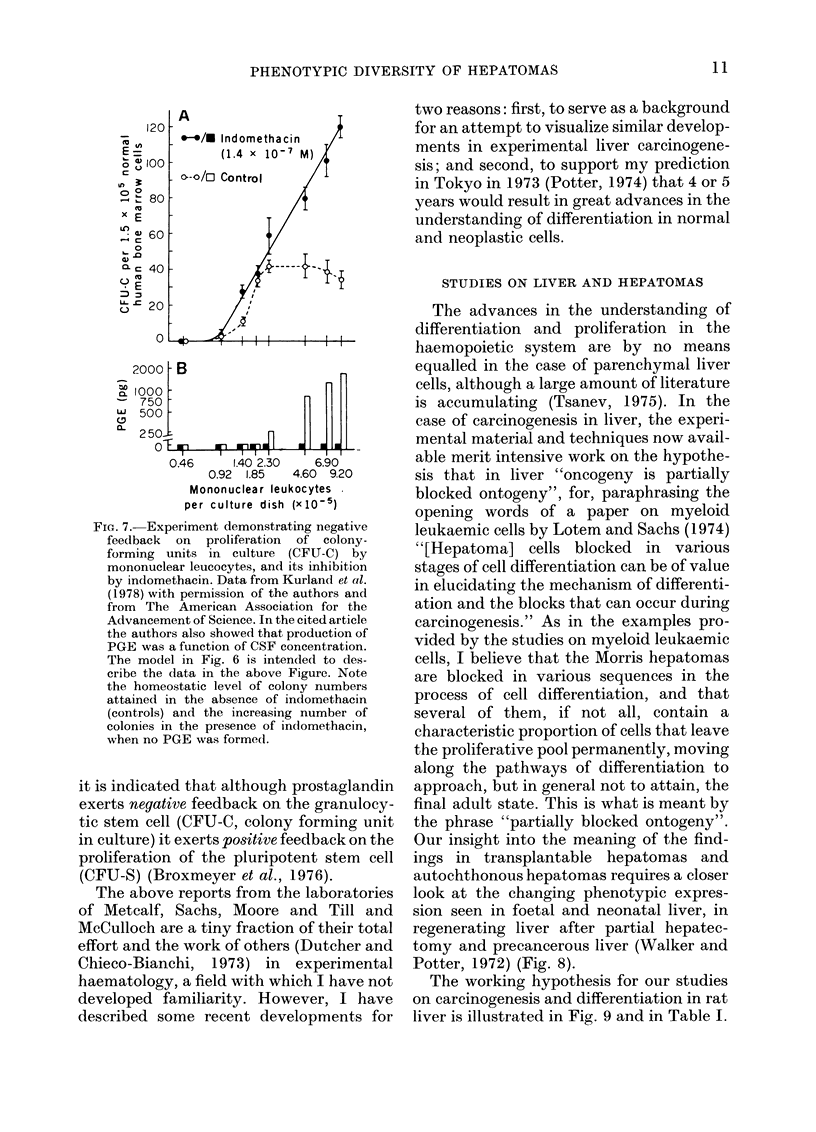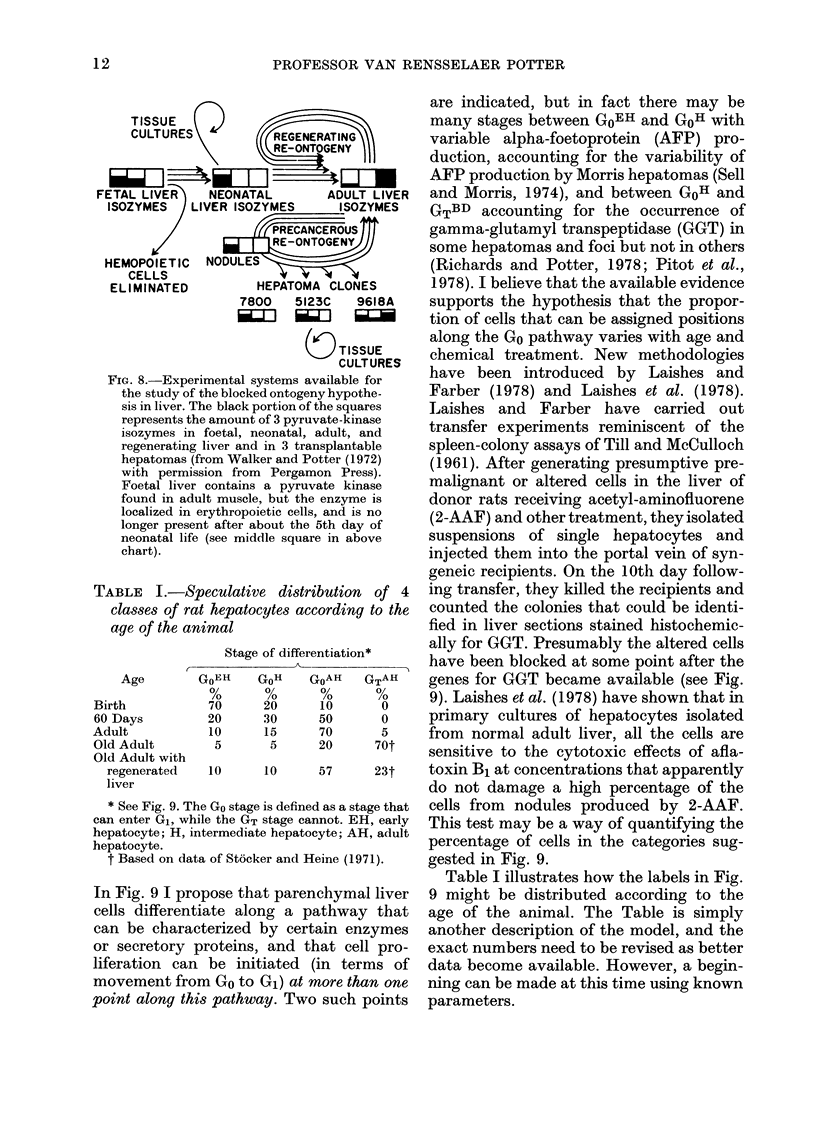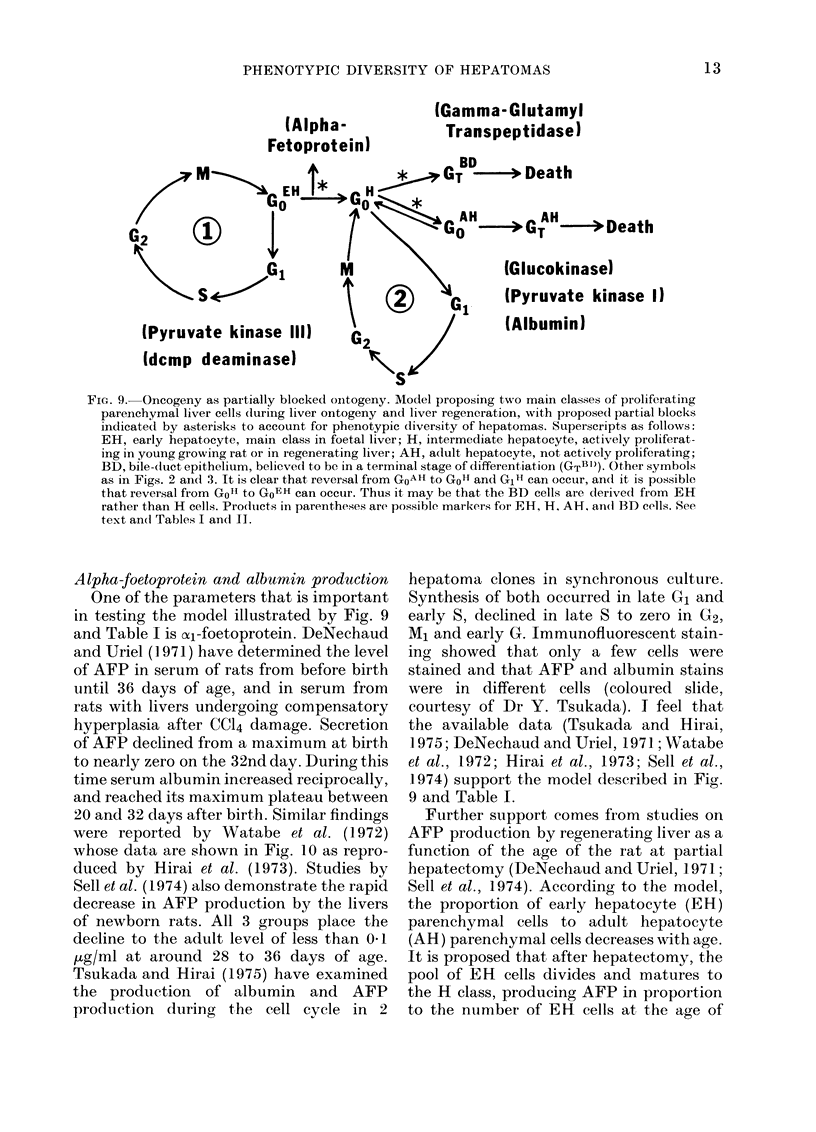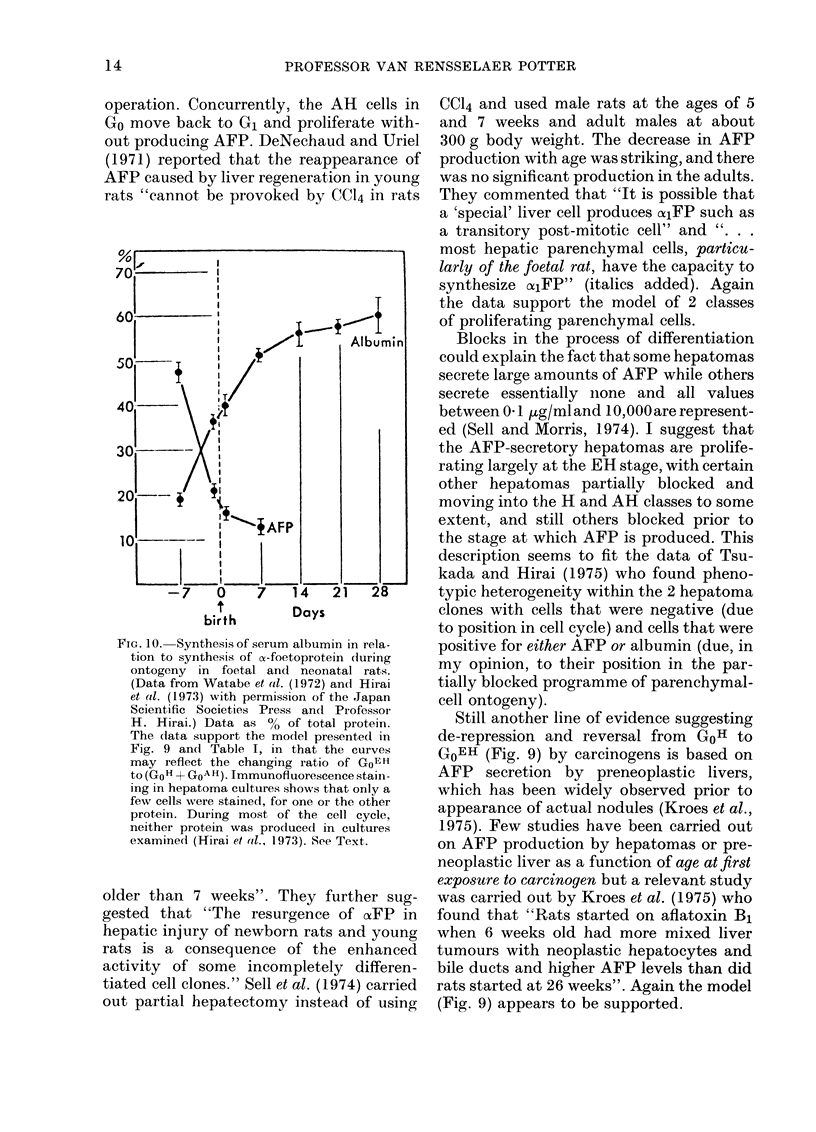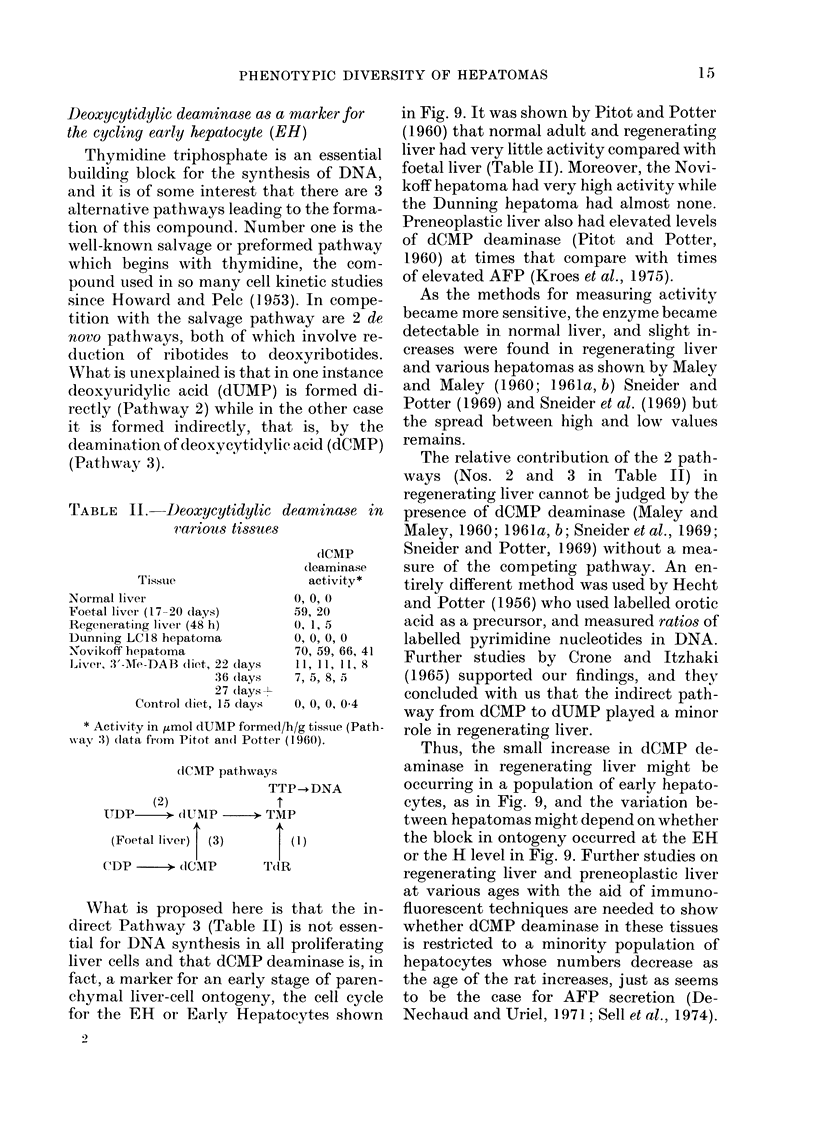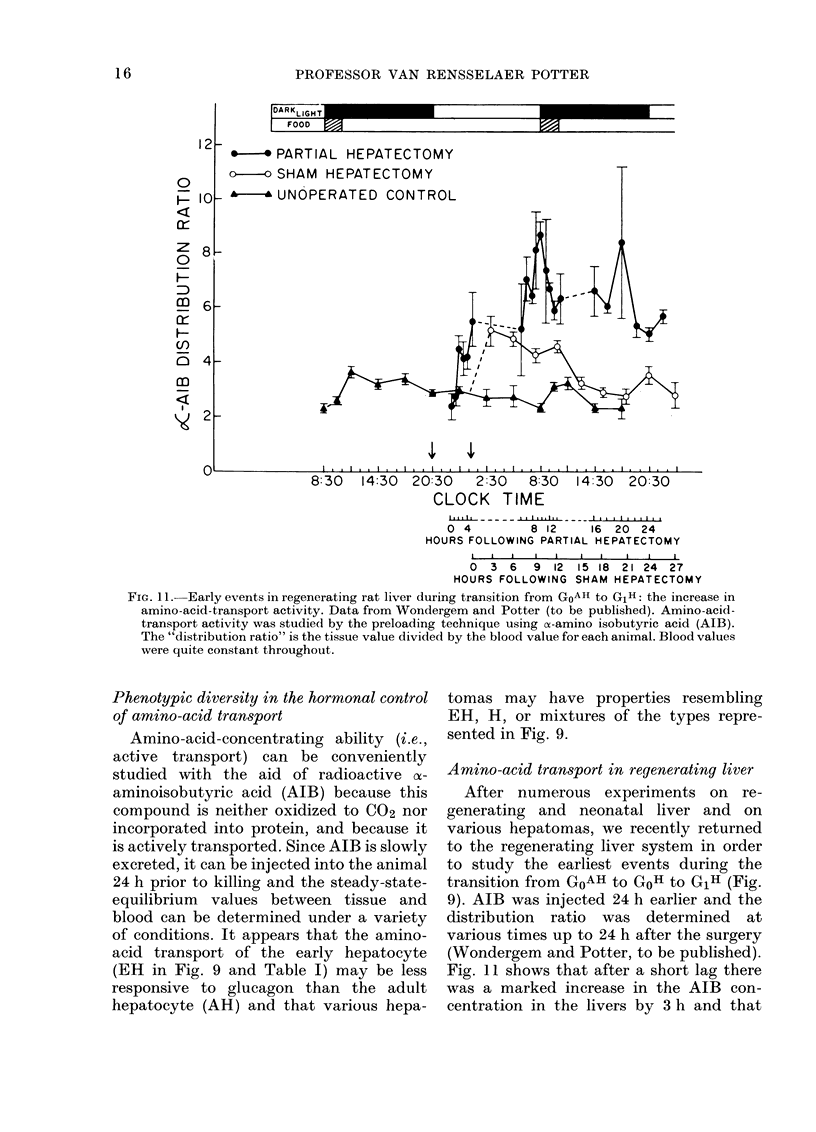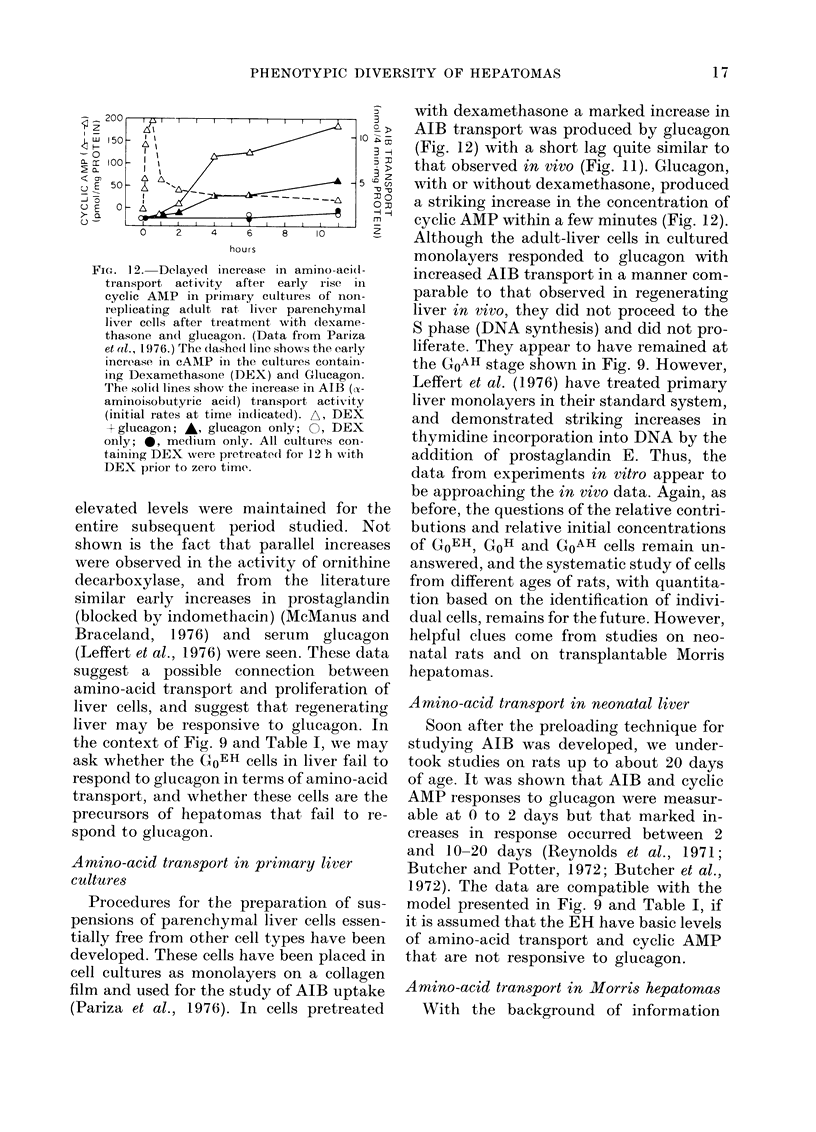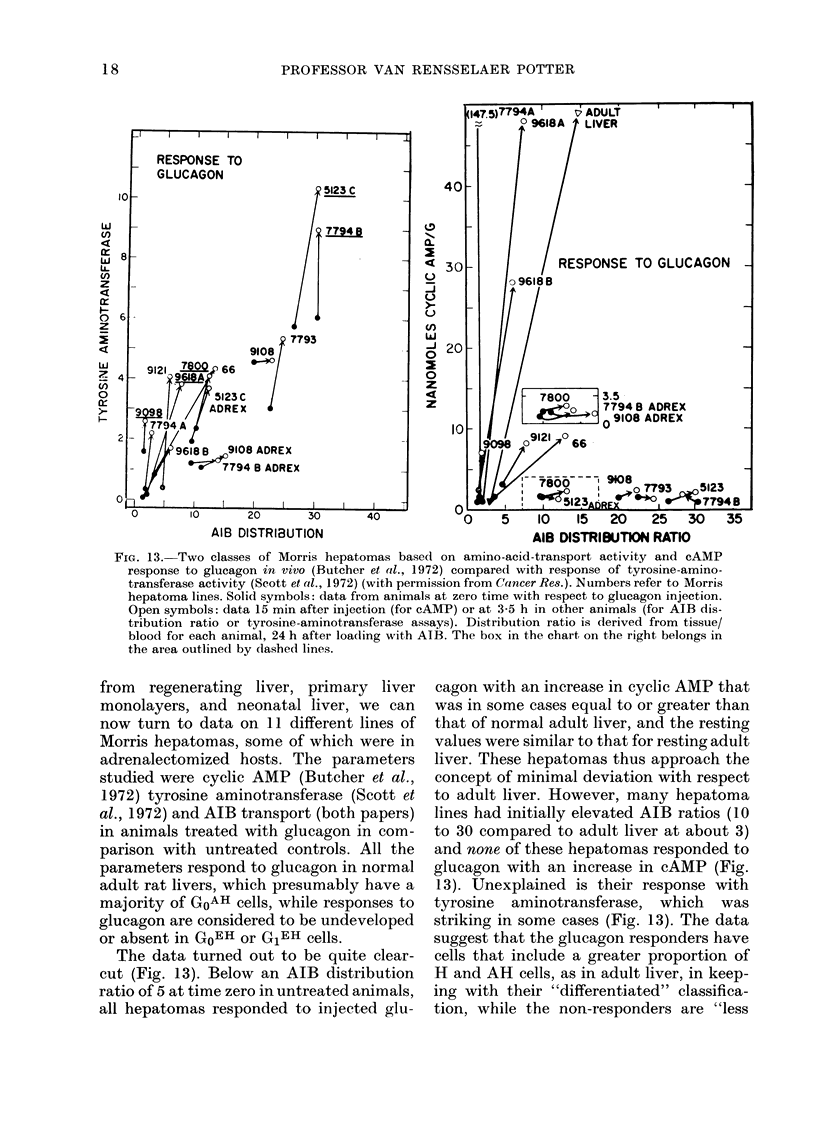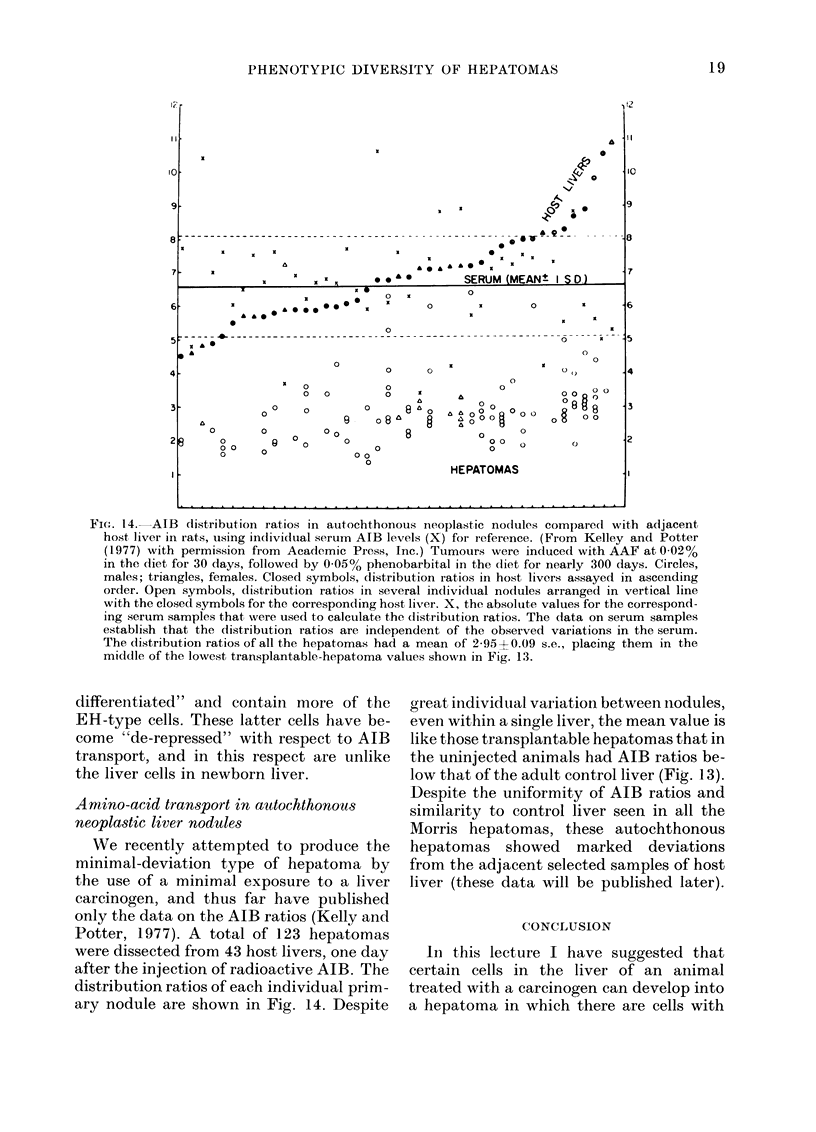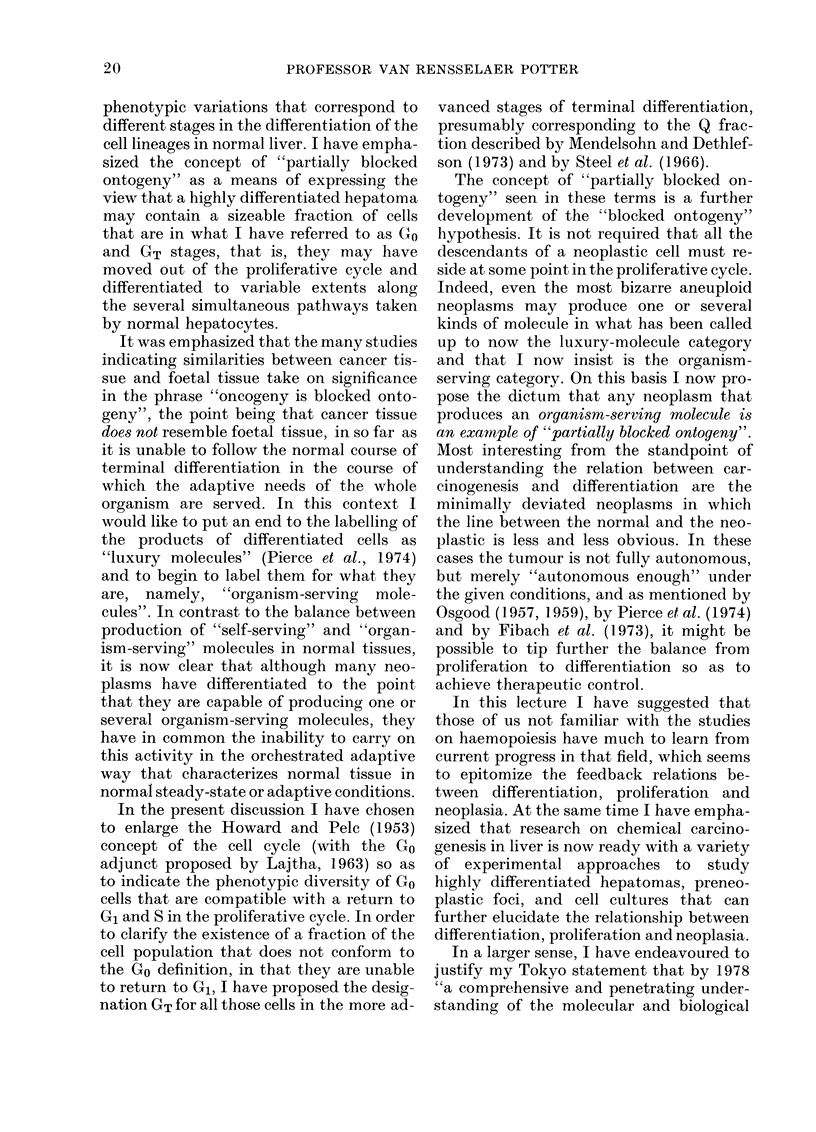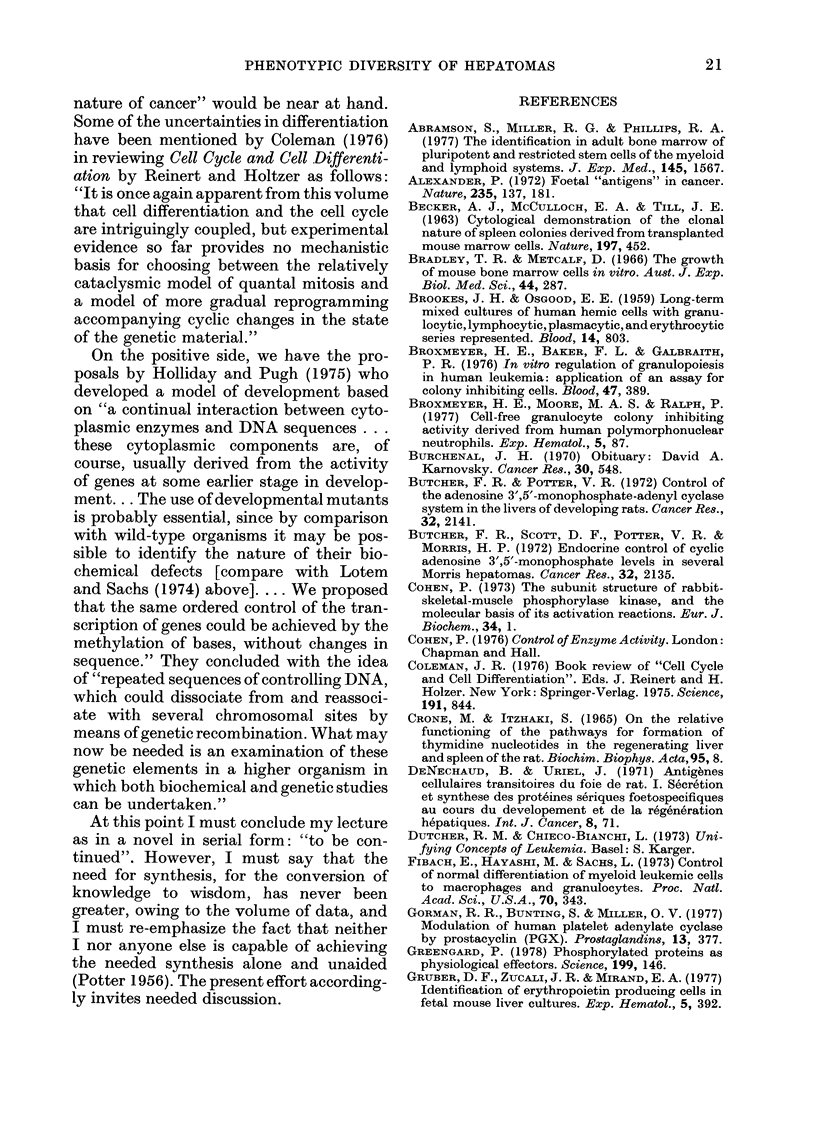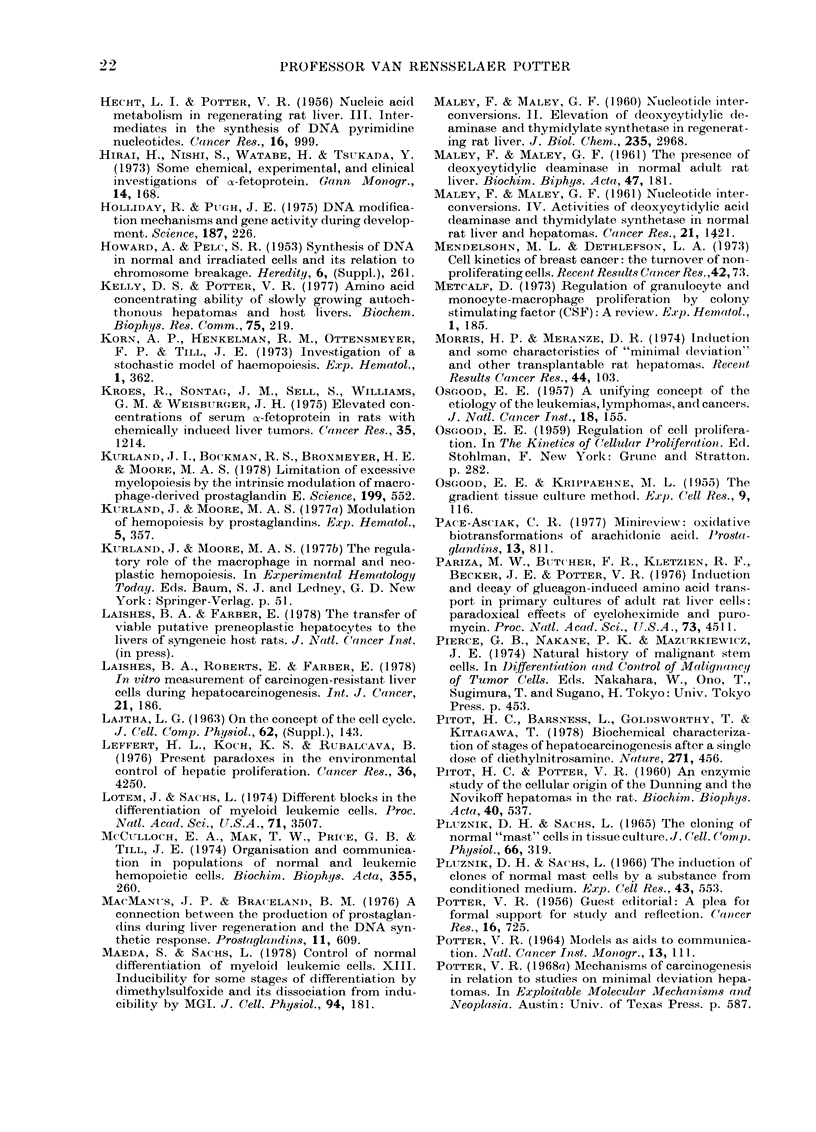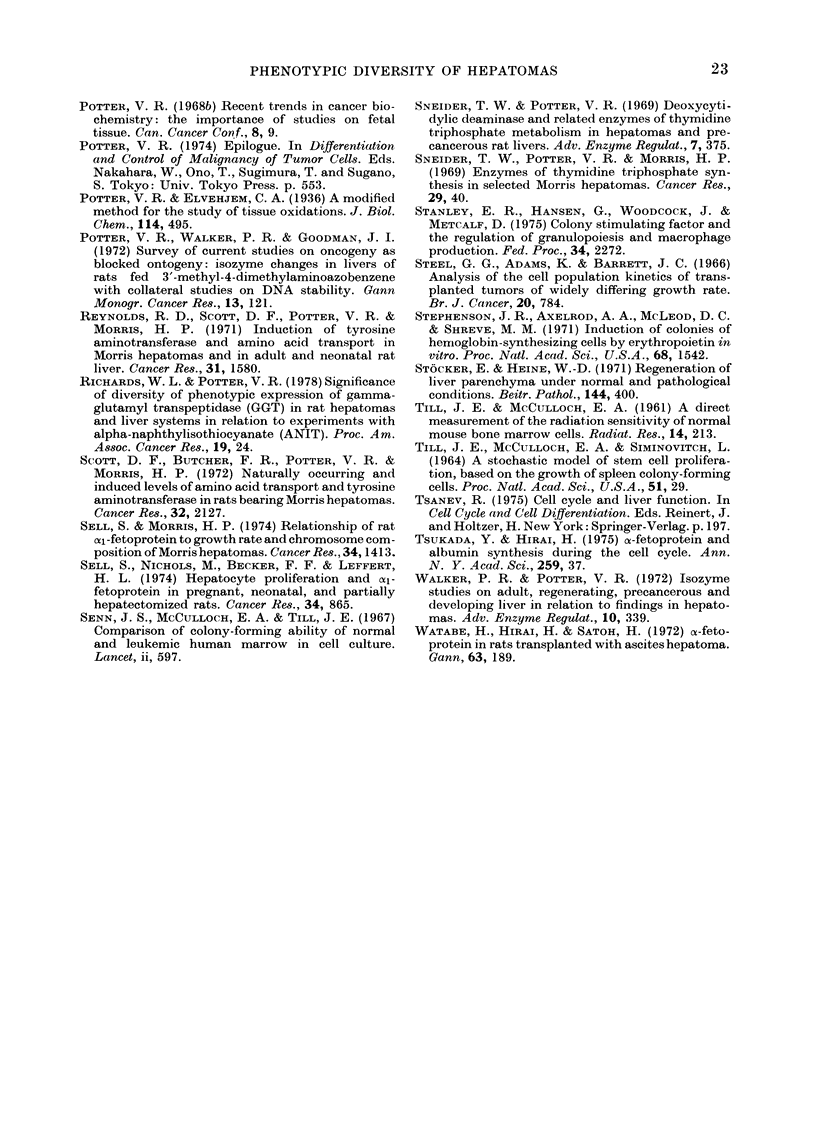# Phenotypic diversity in experimental hepatomas: the concept of partially blocked ontogeny. The 10th Walter Hubert Lecture.

**DOI:** 10.1038/bjc.1978.159

**Published:** 1978-07

**Authors:** V. R. Potter

## Abstract

Cancer cells should be seen not as exclusively a problem in cell proliferation, but rather as a problem combining the processes of proliferation and differentiation, hence the phrase introduced in 1968: "oncogeny is blocked ontogeny". Cancer tissues resemble foetal tissues in many ways but they differ from foetal tissue in being unable to "recapitulate the total programme leading to an orchestrated collection of organism-serving cells" that are programmed "to make the organ as adaptive as possible to the range of environmental variations in which it evolved". Citing the "Osgood Principle" from the 1950's, recent supporting evidence was described, in which the most mature differentiated cells exert positive and negative feedback upon the proliferation of their progenitor stem cells. Advanced examples in the haemopoietic series were drawn from the work of Sachs, Metcalf, Till and McCulloch, and Kurland and Moore. The blocked ontogeny hypothesis was further elaborated in the concept of "partially-blocked ontogeny", which is intended to describe a situation in which highly differentiated slowly growing tumours contain some cells which have left the proliferating pool to differentiate along the normal pathway, but are blocked somewhere short of the final organism-serving state, in harmony with earlier suggestions by Osgood, by Pierce, and by Sachs.


*Br. J. Cancer* (1978) **38**, 1

### PHENOTYPIC DIVERSITY IN EXPERIMENTAL HEPATOMAS: THE CONCEPT OF PARTIALLY BLOCKED ONTOGENY

THE 10th WALTER HUBERT LECTURE
BRITISH ASSOCIATION FOR CANCER RESEARCH, OXFORD, APRIL 1978

PROFESSOR VAN RENSSELAER POTTER

*McArdle Laboratory, University of Wisconsin Medical School, Madison, Wisconsin* 53706, *U.S.A.*

Received 7 April 1978

**Summary.—Cancer cells should be seen not as exclusively a problem in cell proliferation, but rather as a problem combining the processes of proliferation and differentiation, hence the phrase introduced in 1968: "oncogeny is blocked ontogeny". Cancer tissues resemble foetal tissues in many ways but they differ from foetal tissue in being unable to "recapitulate the total programme leading to an orchestrated collection of organism-serving cells" that are programmed "to make the organ as adaptive as possible to the range of environmental variations in which it evolved". Citing the "Osgood Principle" from the 1950's, recent supporting evidence was described, in which the most mature differentiated cells exert positive and negative feedback upon the proliferation of their progenitor stem cells. Advanced examples in the haemopoietic series were drawn from the work of Sachs, Metcalf, Till and McCulloch, and Kurland and Moore. The blocked ontogeny hypothesis was further elaborated in the concept of "partially-blocked ontogeny", which is intended to describe a situation in which highly differentiated slowly growing tumours contain some cells which have left the proliferating pool to differentiate along the normal pathway, but are blocked somewhere short of the final organism-serving state, in harmony with earlier suggestions by Osgood, by Pierce, and by Sachs.**

PHENOTYPIC diversity among cancers is expressed in terms of variable rates of cell proliferation, variable degrees of differentiation, and variation in the metabolic pathways that make proliferation possible. Thus, phenotypic diversity increases the complexity of the cancer problem immeasurably. There is little doubt that if all cancers grew at the same rate and used the same metabolic pathways, the problem would have been solved by now.

Phenotypic diversity in normal tissues may occur by two mechanisms that can be thought of as representing the extremes of the range of possibilities: (1) in the evolution of species, diversity arises by adaptive mutations in the DNA complement that makes up the genotype, and (2) phenotypic diversity can arise through ontogeny, the total programme of differentiation, which has been accepted as a process that alters *the availability but not the information content of the total DNA complement.* Whether the assumption of differentiation without alteration of the DNA sequences (mechanism *2*) can be maintained is still a matter of speculation. In any case, we can benefit by attempting to explain the phenotypic diversity that occurs among those cancers that have incurred the least possible change in the genome. Among the more than 40 Morris hepatomas there are several that have the normal diploid number of chromosomes, though it is not claimed on that basis that the genome has not been altered (Potter, 1968*a*, *b*).

I suggest that phenotypic diversity among cancers can be explained without the need to involve extensive change in the genotype, although change certainly occurs during the natural history of neoplastic disease when, for example, drug-sensitive tumours become resistant. Even in the absence of extensive genetic alteration, I believe that there are a multitude of stages in differentiation, prior to the terminal stage, that, if prevented from advancing toward the terminal stage, will cause a cell to retain its capacity to re-enter the proliferative cycle. Thus, vast phenotypic diversity may be explained by the occurrence of only one or a few genetic alterations that can be effective in blocking differentiation in one or several steps, with results that are compatible with return to proliferation. Along with many others, I believe that cancer is a disease of differentiation.

The problem reduces to what I have referred to (1968*a*) as the "minimal deviation" concept, which, in turn, can be expressed in the framework of the process of differentiation in the phrase "oncogeny is blocked ontogeny" (Potter, 1968*a*, *b*; Potter *et al.*, 1972), a phrase that Alexander (1972) felt was no more than a restatement of the "many similarities between foetal and cancer tissue". The phrase is, however, intended to be much more than a restatement of similarities. The point is not that cancer tissue resembles foetal tissue in some respects, it is that in important ways cancer tissue *does not* resemble foetal tissue. The difference lies in the word *blocked*. An organ from a foetal or newborn animal consists of a population of cells that are participating in an organized and orchestrated programme which takes them through a sequence of changes that are constantly tailored to make the organ as adaptive as possible to the range of environmental variation in which it evolved. In contrast, although a clonal neoplasm that develops from a cell in a particular organ may differentiate into a population that has much of the phenotypic variation seen in a population of normal cells from the same organ, it will not recapitulate the total programme leading to an orchestrated collection of organism-serving cells, because, in my earlier words, the cancer is a case of "blocked ontogeny", and I mean to imply that the total programme of normal ontogeny is blocked somewhere because of a change in one or more chromosomal or extrachromosomal DNA sequences in the original cell from which the neoplastic clone was derived. The concept of blocked ontogeny does not require that all the descendants of the clone must bear the genotypic defect, nor does it require that the block in ontogeny is necessarily at the embryonic or foetal stage of ontogeny for the tissue of origin. The block could conceivably come at *any* point between the dividing cell and the terminal stage of differentiation. It must be clear that the concept of blocked ontogeny has to include "early-blocked ontogeny", that is, exponential proliferation of stem cells that can give rise only to two cells both of which continue to proliferate without further differentiation. In addition, the concept must include "partially blocked ontogeny" in which a substantial *fraction* of the tumour cell *population* undergoes extensive differentiation but does not progress to the normal organized terminal state.

This concept was stated by me (Potter, 1968*b*) as follows: "There may be an extremely large number of intermediate stages between the committed hepatocyte and the adult hepatocyte, wherein a mutant gene could result in a blocked development and produce a clone of cells with a normal chromosome number, but with a propensity for continued cell replication, and with a resistance toward normal feedback controls so that the observer would call it a cancer." At that time it was also stated: "We have now reached a stage in experimental cancer research where it is possible to work with a variety of 42-chromosome* hepatomas and to show that they have diverse phenotypes. We feel that it is feasible to attempt to rationalize the diversity that occurs at this stage of trans-

* The normal diploid number in the rat.

formation. The experimental task need not be the rationalization of the bizarre phenotypes that may appear where cancer cells progress to highly aneuploid genotypes, in which any kind of a non-essential change can occur so long as it permits the cancer cell to continue replicating. At that level of random change it would be asking too much to rationalize the meaning of all the enzyme patterns that are compatible with malignancy."

Five years later, in 1973, addressing Princess Takematsu at the conclusion of the Symposium on *Differentiation and Control of Malignancy of Tumor Cells*, I remarked that "a comprehensive and penetrating understanding of the molecular and biological nature of cancer is very near at hand, so near that when we look back 4 or 5 years from now it may be difficult to say exactly which discoveries were most important in gaining this understanding. Later perhaps a clearer perspective will be gained. But I must caution you that understanding is not synonymous with conquering the disease" (Potter, 1974). On that occasion a hint of the present discussion was also indicated (ibid., p. 186).

Today, these 4 or 5 years have passed and I must attempt to justify the two-fold claim that understanding would be near at hand, and that the contributing concepts and experiments would come from more than a few individuals. My method will be to present an extended introduction, in which a number of advances will be mentioned, and to conclude with a brief mention of some work in my own laboratory seen against the background as described.

### PHENOTYPIC DIVERSITY IN 1956

In discussing phenotypic diversity, a useful point of departure is a symposium sponsored by the American Cancer Society in March 1956 entitled: "A Critical Appraisal of the Biochemical Characteristics of Morphologically Separable Cancers" (*Cancer Res.*, **16**, 639–724). It could have had a simpler title: "Differences among Cancers." The report is noteworthy for the extent of the commentaries provided, and for the expressions of points of view at a time that preceded the era of molecular biology as we now know it, indeed, before the insights permitted by the availability of the Morris hepatomas, of which more later.

### *Diversity in pathology*

I was tremendously influenced by a pathologist (Steiner, ibid. p. 681) whose extensive presentation of coloured slides, showing stained microscope sections, was unfortunately not presented or described in the final report. However, the forceful presentation of personal views remains in print. ". . . are neoplasms one basic disease or are they many different diseases? The problem whether the neoplastic state has an essential defect common to all tumours, formerly only of theoretical interest to a few, has become dominant to investigators in human etiological agents, cancer tests, and chemotherapy. . . . Every tumour cell is a composite of normal and abnormal (neoplastic) in varying proportions. They are morphologically recognized as tumour by their abnormalities, but the residual normal features indicate their origin . . . . . . the changes . . . form the basis for tumour diagnosis from stained tissues, even though the chemical interpretations of the materials visualized are only partly known. The quantity and spatial distribution of the chemical constituents appear altered in tumour cells. *No two tumours are ever alike in this regard, unlike normal tissues. Whether this visible abnormality and diversity represents quality as well as quantity of chemicals is not known. It nevertheless illustrates great diversity in tumours*" (italics added).

"On purely clinical grounds it [cancer] shows both great diversity and a unifying principle. . . . Cutaneous, gastric, and uterine cancers appear to be separate diseases. They are diagnosed and treated by different methods and often by different specialists. However, constantly lurking in the physician's mind is an awareness of a unifying basic biological characteristic shared by all tumours, namely, that of a relentless cell proliferation and its disastrous consequences."

To my knowledge Steiner was the first oncologist to have the courage to say in print that "No two tumours are ever alike." Even though he spoke as a pathologist, the inference might be drawn that the statement applied to basic biochemical differences and to therapeutic response.

*Diversity in chemotherapy*

Karnovsky (ibid., p. 698), speaking on "Differences between Cancers in Terms of Therapeutic Responses", concluded his introduction as follows: "It can be concluded at the outset of this talk, and without much thought, that cancers differ greatly among themselves. This categorical conclusion is not a promising one to the therapist but, if true, it is so important that it should be arrived at only at the completion of a laborious analysis. It is this great diversity among cancers, particularly in the unpredictability of the therapeutic response in individual cases and in the invariable development of resistance to treatment in the responsive cases, that I am discussing today." It may be recalled by some that Dr Karnovsky died, a victim of cancer, in 1969 (Burchenal, 1970) at the age of 55. Although the diversity he referred to still presents difficulties to the therapist, the reference to "invariable development of resistance" would have to be qualified, and in fact Dr Karnovsky lived to see some of his own patients in whom tumour cells were apparently all killed by chemotherapy before the tumours could develop resistance.

*Diversity in biochemistry*

Greenstein (ibid., p. 641) presented a biochemical viewpoint beginning with the words: "The great and seemingly insuperable antithesis of unity and variety may serve as the theme of the present discussion." He went on to emphasize unity as he developed the theme of convergence to a uniform pattern of enzymes, a concept that was correctly analysed by Weinhouse (ibid., p. 654) as possibly applicable to late generations of tumour cells after successive transplantations, but not necessarily to primary tumours which could retain many features of their cell of origin. In the following discussion (ibid., p. 658) I presented what amounted to the biochemical definition of phenotype as follows: "We think that tissues can be characterized by the kind and amount of enzymes that they contain . . . It means that the characterization has to be framed in terms of the spatial organization of enzymes in the cell in nuclei, nucleoli, mitochondria, endoplasmic reticulum, RNA granules [today called ribosomes], soluble enzymes, cell and particulate surfaces, and units as yet undiscovered [today add messenger RNA and polysomes]. It means that the characterization has to specify a particular point on the time scale, both in relation to the life of the tissue and relative to changes in the environment [*e.g.*, changes mediated by hormones]. It means that we have to recognize that tissues are not homogeneous with respect to cell types and that [enzyme] values for tissues depend on the relative proportions of cell types and their possible interaction." Coming from the progenitor of the homogenate technique (Potter and Elvehjem, 1936), I think that statement represents some progress in the 20 years, 1936–56, and moreover that it is a viable statement today, 1978, 22 years later. What *has* changed is not only the availability of new techniques (especially for cell culture) and new parameters (cyclic nucleotides, prostaglandins, new peptide hormones, isoenzymes, *etc.*) but new experimental tumours, both primary and transplantable in isologous hosts, in which the properties of the primary tumour tend to be preserved, *e.g.*, the Morris hepatomas (Morris and Meranze, 1974). Most fascinating of all is the description of new enzymes that regulate phenotypic expression. Their presence or absence *per se* is part of phenotypic expression. These enzymes include *e.g.* adenylcyclase, prostaglandin synthe-

FIG. 1.—Modulation of enzyme activity by cascading multistage controls. Modified from Greengard (1978), Cohen (1973, 1976), Gorman *et al.* (1977) and Pace-Asciak (1977). Cyclic AMP is formed through a variety of mechanisms not involving prostaglandins, and there are protein kinases that do not depend on cyclic AMP (Greengard, 1978).

FIG. 2.—The cell cycle as originally proposed by Howard and Pelc in 1953. M = mitosis; $G_1$ = phase after mitosis and before DNA synthesis; S = phase during which DNA is synthesized, indicated by incorporation of labelled thymidine (marked by asterisk); $G_2$ = phase between S and M.

tase (several components), the "newer" enzymes, prostacyclin synthetase and thromboxane $A_2$ synthetase, all of which are early components of multistage controls, plus the late-stage controls known as protein kinases and protein phosphatases. The literature describing *all* the advances in the biochemical regulation of phenotype expression is too vast to be cited at this point. Greengard (1978), Cohen (1973, 1976), Gorman *et al.* (1977) and Pace-Asciak (1977) are a few examples. However, the particular mechanisms shown in Fig. 1 are relevant to the feedback systems to be discussed later.

### THE EVOLUTION OF THEORY AND EXPERIMENT SINCE 1956

### *The cell cycle*

To support the claim that understanding of the nature of cancer is near at hand, and to single out the names of "more than a few" individuals, is a task that is worthy of a Hubert Lecturer, but one that I approach with trepidation and humility. I have observed that, perhaps more than in any other field, the bibliographies of reviews of cancer research are studded with references to the author's work, usually excluding many references that others might think pertinent. I will necessarily omit many names, but none intentionally, and perhaps on another occasion I could improve over the present effort.

In attempting to cover some aspects of the last 20 years, I will describe models of cell replication and differentiation. In my opinion, models serve as "aids to communication" entirely apart from their relation to reality (Potter, 1964).

The first model of the cell cycle was that of Howard and Pelc (1953) who originated the well-known model based on the incorporation of radioactive thymidine into DNA (Fig. 2).

It was not until 1963 that Lajtha, reasoning from a knowledge of events in regenerating liver, introduced the concept of another stage ($G_0$) appended to the cell cycle. Fig. 3 is developed from his suggestion. The $G_0$ concept was introduced to account for the fact that a period of about 16 to 18 h elapses after partial hepatectomy in rats before the hepatocytes begin to enter the S phase (DNA synthesis). In an adult rat the great majority of the liver cells are not proliferating, and Lajtha proposed the term $G_0$ in preference to assuming that the cells are merely in a prolonged

FIG. 3.—The cell cycle of Fig. 2, with addition of 2 new terms ($G_0$ and $G_T$) directly related to differentiation. $G_0$ was a term introduced by Lajtha in 1963 to indicate a condition distinguished from $G_1$ in that the cell is not preparing for DNA synthesis. Here $G_0$ is defined as any stage of differentiation, up to and including the *n*th stage, provided that the cell can still return to $G_1$ and complete a cycle of proliferation at the same level of differentiation or at a more specialized level. The $G_0$ stage at any level could undergo further specialization until it reached a terminal stage ($G_T$) at which it could no longer return to $G_1$. The $G_T$ cell is conceptually a $G_0{}^{n+1}$ cell in the sense that the $G_0{}^{nth}$ is the last cell in the $G_0$ series that can return to a $G_1$ stage. Rates $k_1$ and $k_2$ are included for comparison with Fig. 4. However, in the present chart, and particularly in regenerating liver, in which events begin from $G_0$, $k_1$ might apply to $G_0 \rightarrow G_1$ and $k_2$ might apply to $G_0 \rightarrow G_T$.

$G_1$ stage of the cell cycle. Essentially 100% of the hepatocytes in a young adult rat (*e.g.*, 90 days old) are capable of returning from $G_0$ to $G_1$, S, $G_2$ and mitosis. In an old rat (*e.g.*, at 2·5 years) this is not the case, as it has been estimated by Stöcker and Heine (1971) that as many as 69% of the hepatocytes cannot return to $G_1$. Thus, it seems appropriate to me to integrate the $G_0$ concept into the cell cycle as shown in Fig. 3, which depicts the $G_0$ stage as a non-proliferating cell that passes through intermediate stages of differentiation, that eventually culminate in a stage of terminal differentiation that I have labelled $G_T$ to indicate that, unlike cells in $G_0$, it cannot return to $G_1$ and the proliferating pool, and that it culminates in the death of the cell. In this way I wish to restrict the definition of a $G_0$ cell by defining it as a cell that can, regardless of its other properties, return to the $G_1$ stage. There must be many diverse cells in $G_0$ that are capable of returning to $G_1$, and the $G_T$ fraction provides for those that cannot. For comparison, I also show a model (Fig. 4) used by Mendelsohn and Dethlefson (1973) and by Steel *et al.* (1966) to describe tumour growth. This latter model seems to make no provision for the occurrence of a $G_0$ tumour cell.

Although non-proliferating, the $G_0$ cell of, say, a young adult mammal can be defined as a cell that has 3 options: (1) it can return to the $G_1$ stage and re-enter the proliferative cycle, (2) it can undergo further differentiation (development?) to new $G_0$ states, by making new parts of its genome available, or (3) it can remain in the same $G_0$ state but with variable production [phenotypic expression] of the so-called luxury molecules in response to organismic demands. Of these possibilities that which provides the best agreement with the concept of differentiation and its role in the development of neoplasia is possibly (2), the maturation to a biochemically or morphologically new $G_0$ that can still re-enter the proliferating cycle. Indeed, instead of thinking of 3 discrete classes of behaviour, it would be more appropriate to inquire how much time a $G_0$ cell in any given series spends in a phenotypically steady state before switching to proliferation or to greater specialization, and to what extent these activities can occur concurrently. Recent advances in the understanding of the control mechanisms involved in the regulation of proliferation *vis-a-vis* differentiation have come from studies of the haemopoietic system.

FIG. 4.—Simplified kinetic model of tumour growth. A model presented by Mendelsohn and Dethlefson (1973). See also Steel *et al.* (1966). P is the proliferating pool of cells, Q the non-proliferating pool, and $k_1$, $k_2$, $k_3$ and $k_4$ are rate constants. The model appears not to provide for $G_0$ cells in the Q pool, *i.e.* for a conversion of non-proliferating cells to the cell cycle as in Fig. 3.

*Spleen-colony-forming cells*

A new line of research began when Till and McCulloch (1961) were able to show that the injection of suspensions of single cells from mouse marrow into heavily irradiated mice produced macroscopic colonies of cells in the spleen at the rate of about 1 per $10^4$ injected. The colonies were shown to be clonal in nature; that is, derived from a single cell (Becker *et al.*, 1963; Abramson *et al.*, 1977). In 1964, further studies by the Toronto group proposed a stochastic model for the early events in a spleen colony, to explain how a clone from a single cell could develop into all the known components of the haemopoietic system (Till *et al.*, 1964). Nine years later, they reaffirmed their original proposition (Korn *et al.*, 1973). According to the stochastic model: "The decision of pluripotent stem cell (S) to self renew, differentiate to the erythroid line (E) or differentiate to the precursor of the granulocyte line (G) is a random event determined by the 3 probabilities $p_S$, $p_E$ and $p_G$ respectively" (Korn *et al.*, 1973). While this model does not include feedback controls it was not developed for the steady-state conditions, since the early events in the spleen colony occur in a heavily irradiated animal, in which cells capable of exerting feedback have been greatly depleted. The stochastic

FIG. 5.—Stochastic early events in a clone from a spleen colony-forming unit (CFU-S) with no feedback indicated, modified from Korn *et al.* (1973) using notations from Figs. 2 and 3. Superscripts S, E, G, MO, and MA indicate CFU-S, erythroid line, granulocytic line, monocyte and macrophage respectively. Initial probabilities indicated by $p_S$, $p_E$ and $p_G$. In addition, the probability concept is extended to indicate two options for every cell in $G_0$: return to $G_1$ or advance toward $G_T$.

model represents the built-in probabilities under whatever feedback may exist in the irradiated animal, and it is understood that the probabilities change as feedback takes over the direction of the outcome. Thus, it was noted that "even though at late times erythroid cells are in the great majority in the colonies, the decision to differentiate to the granulocyte line is favoured over the erythroid line by a ratio of about 10 to 1". Fig. 5 is a representation of the stochastic model modified from that presented by Korn *et al.* (1973) by relating it more closely to Figs. 2 and 3.

At the time of their report, the Toronto authors commented that "the direct study *in vivo* of the early events of a differentiating system, such as haemopoiesis, is not feasible at present. Cells or cell lines are not immediately recognized or identified by morphological or functional criteria as being committed to develop in certain directions, but must propagate and differentiate to a stage where such identification becomes possible" (Korn *et al.*, 1973). Further evolution of the experimental approach suggests that early changes may soon be visualized in individual cells by the techniques of immunofluorescence (Tsukada and Hirai, 1975) with concomitant autoradiographic visualization of cells in which DNA is labelled by $^3$H-thymidine (Howard and Pelc, 1953; Lajtha, 1963; Stöcker and Heine, 1971; Mendelsohn and Dethlefson, 1973; Steel *et al.*, 1966). Remarkable increases in the understanding of the haemopoietic system were made possible by the development of cell culture techniques.

*Colony-forming units studied by cell-culture techniques*

In 1965–67 as reviewed by Metcalf (1973) and by McCulloch *et al.* (1974) 3 groups began the study of the differentiation of the pluripotent stem cell from marrow by cell-culture techniques. These developments occurred in the laboratories of Pluznik and Sachs (1965, 1966) in Israel, Bradley and Metcalf (1966) in Australia and Senn *et al.* (1967) in Canada. In the beginning of the cell-culture studies, the erythroid line, which had developed nicely in the spleen colonies, failed to develop in the culture dishes. However, in 1971 this deficiency was corrected by the Axelrod group (Stephenson *et al.*, 1971). The development of cell-culture techniques made it possible to show that the stochastic model of cell differentiation is overlaid by several layers of both positive and negative feedback controls.

*The Osgood Principle*

Recent developments in the feedback control of haemopoiesis represent the confirmation of what I should like to call the Osgood Principle, which was first formulated in 1950 and reviewed by Osgood in 1957 and 1959 with references to literature on both inhibitors and stimulators. The late E. E. Osgood was a haematologist, whose unique cell-culture method was published in 1955 (Osgood and Krippaehne, 1955). He stated (Osgood, 1957) the principle "that with the majority of cell series *the homeostatic regulator is an inhibitor of arithmetic cell division and is probably produced by the most chemically mature of the differentiating cells of that series*" (italics added). This most chemically mature cell is the cell that I label $G_T$ in Fig. 3. Osgood continued: "somewhat earlier in the process of differentiation an inhibitor of the logarithmic division that constitutes the growth process must be produced. If this were the case, all that would be necessary for unlimited growth—in other words, malignancy–would be the absence of the inhibitor of logarithmic growth. The simplest mechanism which could explain this decreased production of inhibitor would be the early death of the differentiating cell before it reached the stage at which the inhibitor was produced." This seems to be another way of saying that "oncogeny is blocked ontogeny" (Potter, 1968*b*). In both reviews Osgood reasoned that "the inhibitor must be relatively unstable" and "only the cells immediately subjacent are affected, indicating that the inhibitor must be relatively unstable . . ." This emphasis on unstable inhibitors will be referred to later in this section. Osgood designated the stem cell $\alpha$ and the next stage of differentiation an *n* cell, pointing out that the $\alpha$ cell could go to 2 $\alpha$s or 1 $\alpha+1$ *n* cell, while the *n* cell could only produce 2 *n* cells or further differentiate with or without division. In Fig. 3, I equate the $G_0$ cell with Osgood's *n* cell, and wish to suggest that one has to consider the fate of a *population* of $G_0$ cells, in the sense that from a combination of individual possibilities ($2G_1$, $2G_0$ or $1G_0+1G_1$) an outcome of X% $G_0$ and 100—X% $G_1$ would be obtained.

Relating the Osgood Principle to the cancer problem, a final quote merits attention. It was proposed that "any alteration of the genetic material in the somatic alpha cell of a series which leads to early death of the corresponding *n* cell [and I would add: "or which leads to a failure to mature to the $G_T$ stage"] may result in a spectrum of neoplasms of corresponding cellular type, from the most acute, rapidly progressive malignant tumour to the most slowly growing benign tumour depending on how early in the life span of the *n* cell death [or a block] occurs" (Osgood, 1959). This prediction has been illustrated by the case of the Morris hepatomas, since they represent a wide spectrum from poorly to highly differentiated types in which phenotypic diversity may be related to the concept of partially blocked ontogeny. Osgood developed a method for "long-term mixed cultures of human hemic cells" (Osgood and Krippaehne, 1955; Brookes and Osgood, 1959) but he never had access to the modern approaches involving cells embedded in soft agar and underlaid by feeder layers of different cell types that produce specific stimulators and inhibitors. However, he visualized a therapeutic approach based on his principle in the statement: "Only the replacement of the normally gene-produced missing factors necessary for a normal life span [*i.e.*, maturation to $G_T$] of the *n* cell, . . . would lead to a real control of a malignant neoplasm." This view has also been expressed by Pierce *et al.* (1974): "Rather than kill malignant stem cells, it is proposed that studies should be undertaken to direct their differentiation to the benign state" and by Sachs: "A tissue culture line of mouse myeloid leukaemia cells can be induced to form colonies with normal differentiation to mature macrophages and granulocytes by [conditioned medium] that contains MGI [a protein inducer also known as CSF, colony stimulating factor]. . . . Treatment with MGI may thus be of potential value in the therapy of leukaemia", Fibach *et al.*, 1973. Further studies by Sachs have continued to the most recent number XIII under the general heading "Control of Normal Differentiation of Myeloid Leukaemic Cells" (Maeda and Sachs, 1978). Elsewhere Sachs stated: "Malignant cells *blocked in various stages of cell differentiation* can be of value in elucidating the mechanism of differentiation and the *blocks that can occur during carcinogenesis*. We have shown that some, but not all, undifferentiated mammalian myeloid leukaemic cells can be induced to undergo differentiation to mature macrophages and granulocytes . . ." (Lotem and Sachs, 1974) (italics added). It is clear that the phrase "partially blocked ontogeny" and the concept of neoplastic variation all the way from complete to partially blocked ontogeny is the working hypothesis of Sachs and his colleagues, who owe me no debt in this regard.

*Positive feedback controls of colony formation in the haemopoietic system in cell cultures*

As mentioned earlier, the study of colony formation in cell cultures coincided with the demonstration of stimulating factors produced by differentiated cells near the terminal point and exerting positive feedback on the earliest progenitors. Metcalf (1973) has pointed out that "Colony formation *in vitro* by both granulocytic and macrophage cells is wholly dependent on the presence of adequate concentrations of a specific factor, given the operational name, colony stimulating factor (CSF)." The substance is a glycoprotein containing sialic acid (Stanley *et al.*, 1975) and is apparently identical with the factor called MGI in Fibach *et al.* (1973) or CSA by others. It has been reported that CSF from mouse cells is completely unable to replace human CSF in its action on human target cells (Kurland *et al.*, 1978). The action of CSF is apparently quite analogous to erythropoietin (Gruber *et al.*, 1977) which performs similar functions in promoting the erythroid line.

### *Negative feedback controls on colony formation in cell cultures*

In 1977, Broxmeyer *et al.* at the Sloan Kettering Institute reported the elaboration of colony-inhibiting activity (CIA) by polymorphonuclear neutrophils (PMN) and pointed out that the latter derive from progenitor cells committed to granulocyte and monocyte-macrophage differentiation and stimulated by CSA (CSF, MGI). They visualized a combined positive and negative feedback system designed to promote homeostasis in the whole animal. Broxmeyer *et al.* (1976) referred to studies on leukaemic patients whose PMN failed to elaborate the negatively acting CIA. Within a very short time, colony-inhibiting activity was demonstrated with prostaglandin $E_1$ ($PGE_1$). First came a report by Kurland and Moore (1977*a*) studying the inhibitory action of the pure substance $PGE_1$ which was demonstrably active at $10^{-10}$ M at all concentrations of CSF, while $10^{-5}$ M $PGE_1$ produced *total* inhibition of colony production at all CSF concentrations. This was quickly followed in February this year by a report from Kurland *et al.* (1978) showing a direct relationship between CSF concentration and PGE production. In addition, by using the soft-agar technique, with marrow cells in the upper layer separated by a cell-free middle layer from the mononuclear leucocytes in the lower layer, they were able to demonstrate the homeostatic effect of the combined production of CSF and $PGE_1$, and the release from colony-inhibiting action when indomethacin was apparent and no $PGE_1$ was formed. Fig. 6 is my expression of those relationships using the symbols employed in Figs. 2 and 3. The data reproduced from the 1978 report are shown in Fig. 7. A further report on indomethacin effects on normal and neoplastic cell lines has also appeared (Kurland and Moore, 1977*b*). Indomethacin inhibits the synthesis of prostaglandin endoperoxide and subsequent derivatives (Fig. 1.).

With the reports that in some systems prostacyclin is much more active than $PGE_1$, much less stable and a product of a microsomal metabolism system (Gorman *et al.*, 1977; Pace-Asciak, 1977) further studies with the short-lived prostaglandins may be expected.

Of interest is a total haemopoietic scheme by Kurland and Moore, in which

FIG. 6.—Feedback controls on the proliferation of the stem cell. A model based on the experiments of Kurland *et al.* (1978) (data shown in Fig. 7) using the $G_0$ and $G_T$ symbols from Figs. 3 and 5, and $G_1$, S, $G_2$ and M as in Fig. 2. CSF is colony stimulating factor; for other abbreviations (CSA and MGI) see text. PGE is prostaglandin $E_1$ (Kurland *et al.*, 1978). (+)ve, positive feedback; (−)ve, negative feedback. Indomethacin is an inhibitor of the synthesis of prostaglandin endoperoxides and subsequent products (Fig. 1). Note bidirectional positive feedback by CSF on stem cell and on PGE synthesis in the cells that can produce CSF.

FIG. 7.—Experiment demonstrating negative feedback on proliferation of colony-forming units in culture (CFU-C) by mononuclear leucocytes, and its inhibition by indomethacin. Data from Kurland *et al.* (1978) with permission of the authors and from The American Association for the Advancement of Science. In the cited article the authors also showed that production of PGE was a function of CSF concentration. The model in Fig. 6 is intended to describe the data in the above Figure. Note the homeostatic level of colony numbers attained in the absence of indomethacin (controls) and the increasing number of colonies in the presence of indomethacin, when no PGE was formed.

it is indicated that although prostaglandin exerts *negative* feedback on the granulocytic stem cell (CFU-C, colony forming unit in culture) it exerts *positive* feedback on the proliferation of the pluripotent stem cell (CFU-S) (Broxmeyer *et al.*, 1976).

The above reports from the laboratories of Metcalf, Sachs, Moore and Till and McCulloch are a tiny fraction of their total effort and the work of others (Dutcher and Chieco-Bianchi, 1973) in experimental haematology, a field with which I have not developed familiarity. However, I have described some recent developments for two reasons: first, to serve as a background for an attempt to visualize similar developments in experimental liver carcinogenesis; and second, to support my prediction in Tokyo in 1973 (Potter, 1974) that 4 or 5 years would result in great advances in the understanding of differentiation in normal and neoplastic cells.

### STUDIES ON LIVER AND HEPATOMAS

The advances in the understanding of differentiation and proliferation in the haemopoietic system are by no means equalled in the case of parenchymal liver cells, although a large amount of literature is accumulating (Tsanev, 1975). In the case of carcinogenesis in liver, the experimental material and techniques now available merit intensive work on the hypothesis that in liver "oncogeny is partially blocked ontogeny", for, paraphrasing the opening words of a paper on myeloid leukaemic cells by Lotem and Sachs (1974) "[Hepatoma] cells blocked in various stages of cell differentiation can be of value in elucidating the mechanism of differentiation and the blocks that can occur during carcinogenesis." As in the examples provided by the studies on myeloid leukaemic cells, I believe that the Morris hepatomas are blocked in various sequences in the process of cell differentiation, and that several of them, if not all, contain a characteristic proportion of cells that leave the proliferative pool permanently, moving along the pathways of differentiation to approach, but in general not to attain, the final adult state. This is what is meant by the phrase "partially blocked ontogeny". Our insight into the meaning of the findings in transplantable hepatomas and autochthonous hepatomas requires a closer look at the changing phenotypic expression seen in foetal and neonatal liver, in regenerating liver after partial hepatectomy and precancerous liver (Walker and Potter, 1972) (Fig. 8).

The working hypothesis for our studies on carcinogenesis and differentiation in rat liver is illustrated in Fig. 9 and in Table I.

FIG. 8.—Experimental systems available for the study of the blocked ontogeny hypothesis in liver. The black portion of the squares represents the amount of 3 pyruvate-kinase isozymes in foetal, neonatal, adult, and regenerating liver and in 3 transplantable hepatomas (from Walker and Potter (1972) with permission from Pergamon Press). Foetal liver contains a pyruvate kinase found in adult muscle, but the enzyme is localized in erythropoietic cells, and is no longer present after about the 5th day of neonatal life (see middle square in above chart).

TABLE I.—*Speculative distribution of 4 classes of rat hepatocytes according to the age of the animal*

| | Stage of differentiation* | | | |
|---|---|---|---|---|
| Age | $G_0^{EH}$ | $G_0^{H}$ | $G_0^{AH}$ | $G_T^{AH}$ |
| | % | % | % | % |
| Birth | 70 | 20 | 10 | 0 |
| 60 Days | 20 | 30 | 50 | 0 |
| Adult | 10 | 15 | 70 | 5 |
| Old Adult | 5 | 5 | 20 | 70† |
| Old Adult with regenerated liver | 10 | 10 | 57 | 23† |

* See Fig. 9. The $G_0$ stage is defined as a stage that can enter $G_1$, while the $G_T$ stage cannot. EH, early hepatocyte; H, intermediate hepatocyte; AH, adult hepatocyte.

† Based on data of Stöcker and Heine (1971).

In Fig. 9 I propose that parenchymal liver cells differentiate along a pathway that can be characterized by certain enzymes or secretory proteins, and that cell proliferation can be initiated (in terms of movement from $G_0$ to $G_1$) *at more than one point along this pathway.* Two such points are indicated, but in fact there may be many stages between $G_0^{EH}$ and $G_0^{H}$ with variable alpha-foetoprotein (AFP) production, accounting for the variability of AFP production by Morris hepatomas (Sell and Morris, 1974), and between $G_0^{H}$ and $G_T^{BD}$ accounting for the occurrence of gamma-glutamyl transpeptidase (GGT) in some hepatomas and foci but not in others (Richards and Potter, 1978; Pitot *et al.*, 1978). I believe that the available evidence supports the hypothesis that the proportion of cells that can be assigned positions along the $G_0$ pathway varies with age and chemical treatment. New methodologies have been introduced by Laishes and Farber (1978) and Laishes *et al.* (1978). Laishes and Farber have carried out transfer experiments reminiscent of the spleen-colony assays of Till and McCulloch (1961). After generating presumptive premalignant or altered cells in the liver of donor rats receiving acetyl-aminofluorene (2-AAF) and other treatment, they isolated suspensions of single hepatocytes and injected them into the portal vein of syngeneic recipients. On the 10th day following transfer, they killed the recipients and counted the colonies that could be identified in liver sections stained histochemically for GGT. Presumably the altered cells have been blocked at some point after the genes for GGT became available (see Fig. 9). Laishes *et al.* (1978) have shown that in primary cultures of hepatocytes isolated from normal adult liver, all the cells are sensitive to the cytotoxic effects of aflatoxin $B_1$ at concentrations that apparently do not damage a high percentage of the cells from nodules produced by 2-AAF. This test may be a way of quantifying the percentage of cells in the categories suggested in Fig. 9.

Table I illustrates how the labels in Fig. 9 might be distributed according to the age of the animal. The Table is simply another description of the model, and the exact numbers need to be revised as better data become available. However, a beginning can be made at this time using known parameters.

FIG. 9.—Oncogeny as partially blocked ontogeny. Model proposing two main classes of proliferating parenchymal liver cells during liver ontogeny and liver regeneration, with proposed partial blocks indicated by asterisks to account for phenotypic diversity of hepatomas. Superscripts as follows: EH, early hepatocyte, main class in foetal liver; H, intermediate hepatocyte, actively proliferating in young growing rat or in regenerating liver; AH, adult hepatocyte, not actively proliferating; BD, bile-duct epithelium, believed to be in a terminal stage of differentiation ($G_T^{BD}$). Other symbols as in Figs. 2 and 3. It is clear that reversal from $G_0^{AH}$ to $G_0^{H}$ and $G_1^{H}$ can occur, and it is possible that reversal from $G_0^{H}$ to $G_0^{EH}$ can occur. Thus it may be that the BD cells are derived from EH rather than H cells. Products in parentheses are possible markers for EH, H, AH, and BD cells. See text and Tables I and II.

*Alpha-foetoprotein and albumin production*

One of the parameters that is important in testing the model illustrated by Fig. 9 and Table I is $\alpha_1$-foetoprotein. DeNechaud and Uriel (1971) have determined the level of AFP in serum of rats from before birth until 36 days of age, and in serum from rats with livers undergoing compensatory hyperplasia after $CCl_4$ damage. Secretion of AFP declined from a maximum at birth to nearly zero on the 32nd day. During this time serum albumin increased reciprocally, and reached its maximum plateau between 20 and 32 days after birth. Similar findings were reported by Watabe *et al.* (1972) whose data are shown in Fig. 10 as reproduced by Hirai *et al.* (1973). Studies by Sell *et al.* (1974) also demonstrate the rapid decrease in AFP production by the livers of newborn rats. All 3 groups place the decline to the adult level of less than 0·1 μg/ml at around 28 to 36 days of age. Tsukada and Hirai (1975) have examined the production of albumin and AFP production during the cell cycle in 2 hepatoma clones in synchronous culture. Synthesis of both occurred in late $G_1$ and early S, declined in late S to zero in $G_2$, $M_1$ and early G. Immunofluorescent staining showed that only a few cells were stained and that AFP and albumin stains were in different cells (coloured slide, courtesy of Dr Y. Tsukada). I feel that the available data (Tsukada and Hirai, 1975; DeNechaud and Uriel, 1971; Watabe *et al.*, 1972; Hirai *et al.*, 1973; Sell *et al.*, 1974) support the model described in Fig. 9 and Table I.

Further support comes from studies on AFP production by regenerating liver as a function of the age of the rat at partial hepatectomy (DeNechaud and Uriel, 1971; Sell *et al.*, 1974). According to the model, the proportion of early hepatocyte (EH) parenchymal cells to adult hepatocyte (AH) parenchymal cells decreases with age. It is proposed that after hepatectomy, the pool of EH cells divides and matures to the H class, producing AFP in proportion to the number of EH cells at the age of operation. Concurrently, the AH cells in $G_0$ move back to $G_1$ and proliferate without producing AFP. DeNechaud and Uriel (1971) reported that the reappearance of AFP caused by liver regeneration in young rats "cannot be provoked by $CCl_4$ in rats

FIG. 10.—Synthesis of serum albumin in relation to synthesis of α-foetoprotein during ontogeny in foetal and neonatal rats. (Data from Watabe *et al.* (1972) and Hirai *et al.* (1973) with permission of the Japan Scientific Societies Press and Professor H. Hirai.) Data as % of total protein. The data support the model presented in Fig. 9 and Table I, in that the curves may reflect the changing ratio of $G_0^{EH}$ to $(G_0^{H}+G_0^{AH})$. Immunofluorescence staining in hepatoma cultures shows that only a few cells were stained, for one or the other protein. During most of the cell cycle, neither protein was produced in cultures examined (Hirai *et al.*, 1973). See Text.

older than 7 weeks". They further suggested that "The resurgence of αFP in hepatic injury of newborn rats and young rats is a consequence of the enhanced activity of some incompletely differentiated cell clones." Sell *et al.* (1974) carried out partial hepatectomy instead of using $CCl_4$ and used male rats at the ages of 5 and 7 weeks and adult males at about 300 g body weight. The decrease in AFP production with age was striking, and there was no significant production in the adults. They commented that "It is possible that a 'special' liver cell produces $\alpha_1$FP such as a transitory post-mitotic cell" and ". . . most hepatic parenchymal cells, *particularly of the foetal rat*, have the capacity to synthesize $\alpha_1$FP" (italics added). Again the data support the model of 2 classes of proliferating parenchymal cells.

Blocks in the process of differentiation could explain the fact that some hepatomas secrete large amounts of AFP while others secrete essentially none and all values between 0·1 μg/ml and 10,000 are represented (Sell and Morris, 1974). I suggest that the AFP-secretory hepatomas are proliferating largely at the EH stage, with certain other hepatomas partially blocked and moving into the H and AH classes to some extent, and still others blocked prior to the stage at which AFP is produced. This description seems to fit the data of Tsukada and Hirai (1975) who found phenotypic heterogeneity within the 2 hepatoma clones with cells that were negative (due to position in cell cycle) and cells that were positive for *either* AFP *or* albumin (due, in my opinion, to their position in the partially blocked programme of parenchymal-cell ontogeny).

Still another line of evidence suggesting de-repression and reversal from $G_0^{H}$ to $G_0^{EH}$ (Fig. 9) by carcinogens is based on AFP secretion by preneoplastic livers, which has been widely observed prior to appearance of actual nodules (Kroes *et al.*, 1975). Few studies have been carried out on AFP production by hepatomas or preneoplastic liver as a function of *age at first exposure to carcinogen* but a relevant study was carried out by Kroes *et al.* (1975) who found that "Rats started on aflatoxin $B_1$ when 6 weeks old had more mixed liver tumours with neoplastic hepatocytes and bile ducts and higher AFP levels than did rats started at 26 weeks". Again the model (Fig. 9) appears to be supported.

*Deoxycytidylic deaminase as a marker for the cycling early hepatocyte (EH)*

Thymidine triphosphate is an essential building block for the synthesis of DNA, and it is of some interest that there are 3 alternative pathways leading to the formation of this compound. Number one is the well-known salvage or preformed pathway which begins with thymidine, the compound used in so many cell kinetic studies since Howard and Pelc (1953). In competition with the salvage pathway are 2 *de novo* pathways, both of which involve reduction of ribotides to deoxyribotides. What is unexplained is that in one instance deoxyuridylic acid (dUMP) is formed directly (Pathway 2) while in the other case it is formed indirectly, that is, by the deamination of deoxycytidylic acid (dCMP) (Pathway 3).

TABLE II.—*Deoxycytidylic deaminase in various tissues*

| Tissue | dCMP deaminase activity* |
|---|---|
| Normal liver | 0, 0, 0 |
| Foetal liver (17–20 days) | 59, 20 |
| Regenerating liver (48 h) | 0, 1, 5 |
| Dunning LC18 hepatoma | 0, 0, 0, 0 |
| Novikoff hepatoma | 70, 59, 66, 41 |
| Liver, 3′-Me-DAB diet, 22 days | 11, 11, 11, 8 |
| 36 days | 7, 5, 8, 5 |
| 27 days+ | |
| Control diet, 15 days | 0, 0, 0, 0·4 |

* Activity in μmol dUMP formed/h/g tissue (Pathway 3) data from Pitot and Potter (1960).

dCMP pathways

What is proposed here is that the indirect Pathway 3 (Table II) is not essential for DNA synthesis in all proliferating liver cells and that dCMP deaminase is, in fact, a marker for an early stage of parenchymal liver-cell ontogeny, the cell cycle for the EH or Early Hepatocytes shown in Fig. 9. It was shown by Pitot and Potter (1960) that normal adult and regenerating liver had very little activity compared with foetal liver (Table II). Moreover, the Novikoff hepatoma had very high activity while the Dunning hepatoma had almost none. Preneoplastic liver also had elevated levels of dCMP deaminase (Pitot and Potter, 1960) at times that compare with times of elevated AFP (Kroes *et al.*, 1975).

As the methods for measuring activity became more sensitive, the enzyme became detectable in normal liver, and slight increases were found in regenerating liver and various hepatomas as shown by Maley and Maley (1960; 1961*a*, *b*) Sneider and Potter (1969) and Sneider *et al.* (1969) but the spread between high and low values remains.

The relative contribution of the 2 pathways (Nos. 2 and 3 in Table II) in regenerating liver cannot be judged by the presence of dCMP deaminase (Maley and Maley, 1960; 1961*a*, *b*; Sneider *et al.*, 1969; Sneider and Potter, 1969) without a measure of the competing pathway. An entirely different method was used by Hecht and Potter (1956) who used labelled orotic acid as a precursor, and measured *ratios* of labelled pyrimidine nucleotides in DNA. Further studies by Crone and Itzhaki (1965) supported our findings, and they concluded with us that the indirect pathway from dCMP to dUMP played a minor role in regenerating liver.

Thus, the small increase in dCMP deaminase in regenerating liver might be occurring in a population of early hepatocytes, as in Fig. 9, and the variation between hepatomas might depend on whether the block in ontogeny occurred at the EH or the H level in Fig. 9. Further studies on regenerating liver and preneoplastic liver at various ages with the aid of immunofluorescent techniques are needed to show whether dCMP deaminase in these tissues is restricted to a minority population of hepatocytes whose numbers decrease as the age of the rat increases, just as seems to be the case for AFP secretion (DeNechaud and Uriel, 1971; Sell *et al.*, 1974).

FIG. 11.—Early events in regenerating rat liver during transition from $G_0^{AH}$ to $G_1^{H}$: the increase in amino-acid-transport activity. Data from Wondergem and Potter (to be published). Amino-acid-transport activity was studied by the preloading technique using α-amino isobutyric acid (AIB). The "distribution ratio" is the tissue value divided by the blood value for each animal. Blood values were quite constant throughout.

*Phenotypic diversity in the hormonal control of amino-acid transport*

Amino-acid-concentrating ability (*i.e.*, active transport) can be conveniently studied with the aid of radioactive α-aminoisobutyric acid (AIB) because this compound is neither oxidized to $CO_2$ nor incorporated into protein, and because it is actively transported. Since AIB is slowly excreted, it can be injected into the animal 24 h prior to killing and the steady-state-equilibrium values between tissue and blood can be determined under a variety of conditions. It appears that the amino-acid transport of the early hepatocyte (EH in Fig. 9 and Table I) may be less responsive to glucagon than the adult hepatocyte (AH) and that various hepatomas may have properties resembling EH, H, or mixtures of the types represented in Fig. 9.

*Amino-acid transport in regenerating liver*

After numerous experiments on regenerating and neonatal liver and on various hepatomas, we recently returned to the regenerating liver system in order to study the earliest events during the transition from $G_0^{AH}$ to $G_0^{H}$ to $G_1^{H}$ (Fig. 9). AIB was injected 24 h earlier and the distribution ratio was determined at various times up to 24 h after the surgery (Wondergem and Potter, to be published). Fig. 11 shows that after a short lag there was a marked increase in the AIB concentration in the livers by 3 h and that

FIG. 12.—Delayed increase in amino-acid-transport activity after early rise in cyclic AMP in primary cultures of non-replicating adult rat liver parenchymal liver cells after treatment with dexamethasone and glucagon. (Data from Pariza *et al.*, 1976.) The dashed line shows the early increase in cAMP in the cultures containing Dexamethasone (DEX) and Glucagon. The solid lines show the increase in AIB (α-aminoisobutyric acid) transport activity (initial rates at time indicated). △, DEX + glucagon; ▲, glucagon only; ○, DEX only; ●, medium only. All cultures containing DEX were pretreated for 12 h with DEX prior to zero time.

elevated levels were maintained for the entire subsequent period studied. Not shown is the fact that parallel increases were observed in the activity of ornithine decarboxylase, and from the literature similar early increases in prostaglandin (blocked by indomethacin) (McManus and Braceland, 1976) and serum glucagon (Leffert *et al.*, 1976) were seen. These data suggest a possible connection between amino-acid transport and proliferation of liver cells, and suggest that regenerating liver may be responsive to glucagon. In the context of Fig. 9 and Table I, we may ask whether the $G_0^{EH}$ cells in liver fail to respond to glucagon in terms of amino-acid transport, and whether these cells are the precursors of hepatomas that fail to respond to glucagon.

*Amino-acid transport in primary liver cultures*

Procedures for the preparation of suspensions of parenchymal liver cells essentially free from other cell types have been developed. These cells have been placed in cell cultures as monolayers on a collagen film and used for the study of AIB uptake (Pariza *et al.*, 1976). In cells pretreated with dexamethasone a marked increase in AIB transport was produced by glucagon (Fig. 12) with a short lag quite similar to that observed *in vivo* (Fig. 11). Glucagon, with or without dexamethasone, produced a striking increase in the concentration of cyclic AMP within a few minutes (Fig. 12). Although the adult-liver cells in cultured monolayers responded to glucagon with increased AIB transport in a manner comparable to that observed in regenerating liver *in vivo*, they did not proceed to the S phase (DNA synthesis) and did not proliferate. They appear to have remained at the $G_0^{AH}$ stage shown in Fig. 9. However, Leffert *et al.* (1976) have treated primary liver monolayers in their standard system, and demonstrated striking increases in thymidine incorporation into DNA by the addition of prostaglandin E. Thus, the data from experiments *in vitro* appear to be approaching the *in vivo* data. Again, as before, the questions of the relative contributions and relative initial concentrations of $G_0^{EH}$, $G_0^{H}$ and $G_0^{AH}$ cells remain unanswered, and the systematic study of cells from different ages of rats, with quantitation based on the identification of individual cells, remains for the future. However, helpful clues come from studies on neonatal rats and on transplantable Morris hepatomas.

*Amino-acid transport in neonatal liver*

Soon after the preloading technique for studying AIB was developed, we undertook studies on rats up to about 20 days of age. It was shown that AIB and cyclic AMP responses to glucagon were measurable at 0 to 2 days but that marked increases in response occurred between 2 and 10–20 days (Reynolds *et al.*, 1971; Butcher and Potter, 1972; Butcher *et al.*, 1972). The data are compatible with the model presented in Fig. 9 and Table I, if it is assumed that the EH have basic levels of amino-acid transport and cyclic AMP that are not responsive to glucagon.

*Amino-acid transport in Morris hepatomas*

With the background of information

FIG. 13.—Two classes of Morris hepatomas based on amino-acid-transport activity and cAMP response to glucagon *in vivo* (Butcher *et al.*, 1972) compared with response of tyrosine-aminotransferase activity (Scott *et al.*, 1972) (with permission from *Cancer Res.*). Numbers refer to Morris hepatoma lines. Solid symbols: data from animals at zero time with respect to glucagon injection. Open symbols: data 15 min after injection (for cAMP) or at 3·5 h in other animals (for AIB distribution ratio or tyrosine-aminotransferase assays). Distribution ratio is derived from tissue/blood for each animal, 24 h after loading with AIB. The box in the chart on the right belongs in the area outlined by dashed lines.

from regenerating liver, primary liver monolayers, and neonatal liver, we can now turn to data on 11 different lines of Morris hepatomas, some of which were in adrenalectomized hosts. The parameters studied were cyclic AMP (Butcher *et al.*, 1972) tyrosine aminotransferase (Scott *et al.*, 1972) and AIB transport (both papers) in animals treated with glucagon in comparison with untreated controls. All the parameters respond to glucagon in normal adult rat livers, which presumably have a majority of $G_0^{AH}$ cells, while responses to glucagon are considered to be undeveloped or absent in $G_0^{EH}$ or $G_1^{EH}$ cells.

The data turned out to be quite clear-cut (Fig. 13). Below an AIB distribution ratio of 5 at time zero in untreated animals, all hepatomas responded to injected glucagon with an increase in cyclic AMP that was in some cases equal to or greater than that of normal adult liver, and the resting values were similar to that for resting adult liver. These hepatomas thus approach the concept of minimal deviation with respect to adult liver. However, many hepatoma lines had initially elevated AIB ratios (10 to 30 compared to adult liver at about 3) and *none* of these hepatomas responded to glucagon with an increase in cAMP (Fig. 13). Unexplained is their response with tyrosine aminotransferase, which was striking in some cases (Fig. 13). The data suggest that the glucagon responders have cells that include a greater proportion of H and AH cells, as in adult liver, in keeping with their "differentiated" classification, while the non-responders are "less

FIG. 14.—AIB distribution ratios in autochthonous neoplastic nodules compared with adjacent host liver in rats, using individual serum AIB levels (X) for reference. (From Kelley and Potter (1977) with permission from Academic Press, Inc.) Tumours were induced with AAF at 0·02% in the diet for 30 days, followed by 0·05% phenobarbital in the diet for nearly 300 days. Circles, males; triangles, females. Closed symbols, distribution ratios in host livers assayed in ascending order. Open symbols, distribution ratios in several individual nodules arranged in vertical line with the closed symbols for the corresponding host liver. X, the absolute values for the corresponding serum samples that were used to calculate the distribution ratios. The data on serum samples establish that the distribution ratios are independent of the observed variations in the serum. The distribution ratios of all the hepatomas had a mean of 2·95±0.09 s.e., placing them in the middle of the lowest transplantable-hepatoma values shown in Fig. 13.

differentiated" and contain more of the EH-type cells. These latter cells have become "de-repressed" with respect to AIB transport, and in this respect are unlike the liver cells in newborn liver.

### *Amino-acid transport in autochthonous neoplastic liver nodules*

We recently attempted to produce the minimal-deviation type of hepatoma by the use of a minimal exposure to a liver carcinogen, and thus far have published only the data on the AIB ratios (Kelly and Potter, 1977). A total of 123 hepatomas were dissected from 43 host livers, one day after the injection of radioactive AIB. The distribution ratios of each individual primary nodule are shown in Fig. 14. Despite great individual variation between nodules, even within a single liver, the mean value is like those transplantable hepatomas that in the uninjected animals had AIB ratios below that of the adult control liver (Fig. 13). Despite the uniformity of AIB ratios and similarity to control liver seen in all the Morris hepatomas, these autochthonous hepatomas showed marked deviations from the adjacent selected samples of host liver (these data will be published later).

### CONCLUSION

In this lecture I have suggested that certain cells in the liver of an animal treated with a carcinogen can develop into a hepatoma in which there are cells with phenotypic variations that correspond to different stages in the differentiation of the cell lineages in normal liver. I have emphasized the concept of "partially blocked ontogeny" as a means of expressing the view that a highly differentiated hepatoma may contain a sizeable fraction of cells that are in what I have referred to as $G_0$ and $G_T$ stages, that is, they may have moved out of the proliferative cycle and differentiated to variable extents along the several simultaneous pathways taken by normal hepatocytes.

It was emphasized that the many studies indicating similarities between cancer tissue and foetal tissue take on significance in the phrase "oncogeny is blocked ontogeny", the point being that cancer tissue *does not* resemble foetal tissue, in so far as it is unable to follow the normal course of terminal differentiation in the course of which the adaptive needs of the whole organism are served. In this context I would like to put an end to the labelling of the products of differentiated cells as "luxury molecules" (Pierce *et al.*, 1974) and to begin to label them for what they are, namely, "organism-serving molecules". In contrast to the balance between production of "self-serving" and "organism-serving" molecules in normal tissues, it is now clear that although many neoplasms have differentiated to the point that they are capable of producing one or several organism-serving molecules, they have in common the inability to carry on this activity in the orchestrated adaptive way that characterizes normal tissue in normal steady-state or adaptive conditions.

In the present discussion I have chosen to enlarge the Howard and Pelc (1953) concept of the cell cycle (with the $G_0$ adjunct proposed by Lajtha, 1963) so as to indicate the phenotypic diversity of $G_0$ cells that are compatible with a return to $G_1$ and S in the proliferative cycle. In order to clarify the existence of a fraction of the cell population that does not conform to the $G_0$ definition, in that they are unable to return to $G_1$, I have proposed the designation $G_T$ for all those cells in the more advanced stages of terminal differentiation, presumably corresponding to the Q fraction described by Mendelsohn and Dethlefson (1973) and by Steel *et al.* (1966).

The concept of "partially blocked ontogeny" seen in these terms is a further development of the "blocked ontogeny" hypothesis. It is not required that all the descendants of a neoplastic cell must reside at some point in the proliferative cycle. Indeed, even the most bizarre aneuploid neoplasms may produce one or several kinds of molecule in what has been called up to now the luxury-molecule category and that I now insist is the organism-serving category. On this basis I now propose the dictum that any neoplasm that produces an *organism-serving molecule is an example of "partially blocked ontogeny"*. Most interesting from the standpoint of understanding the relation between carcinogenesis and differentiation are the minimally deviated neoplasms in which the line between the normal and the neoplastic is less and less obvious. In these cases the tumour is not fully autonomous, but merely "autonomous enough" under the given conditions, and as mentioned by Osgood (1957, 1959), by Pierce *et al.* (1974) and by Fibach *et al.* (1973), it might be possible to tip further the balance from proliferation to differentiation so as to achieve therapeutic control.

In this lecture I have suggested that those of us not familiar with the studies on haemopoiesis have much to learn from current progress in that field, which seems to epitomize the feedback relations between differentiation, proliferation and neoplasia. At the same time I have emphasized that research on chemical carcinogenesis in liver is now ready with a variety of experimental approaches to study highly differentiated hepatomas, preneoplastic foci, and cell cultures that can further elucidate the relationship between differentiation, proliferation and neoplasia.

In a larger sense, I have endeavoured to justify my Tokyo statement that by 1978 "a comprehensive and penetrating understanding of the molecular and biological nature of cancer" would be near at hand. Some of the uncertainties in differentiation have been mentioned by Coleman (1976) in reviewing *Cell Cycle and Cell Differentiation* by Reinert and Holtzer as follows: "It is once again apparent from this volume that cell differentiation and the cell cycle are intriguingly coupled, but experimental evidence so far provides no mechanistic basis for choosing between the relatively cataclysmic model of quantal mitosis and a model of more gradual reprogramming accompanying cyclic changes in the state of the genetic material."

On the positive side, we have the proposals by Holliday and Pugh (1975) who developed a model of development based on "a continual interaction between cytoplasmic enzymes and DNA sequences . . . these cytoplasmic components are, of course, usually derived from the activity of genes at some earlier stage in development. . . The use of developmental mutants is probably essential, since by comparison with wild-type organisms it may be possible to identify the nature of their biochemical defects [compare with Lotem and Sachs (1974) above]. . . . We proposed that the same ordered control of the transcription of genes could be achieved by the methylation of bases, without changes in sequence." They concluded with the idea of "repeated sequences of controlling DNA, which could dissociate from and reassociate with several chromosomal sites by means of genetic recombination. What may now be needed is an examination of these genetic elements in a higher organism in which both biochemical and genetic studies can be undertaken."

At this point I must conclude my lecture as in a novel in serial form: "to be continued". However, I must say that the need for synthesis, for the conversion of knowledge to wisdom, has never been greater, owing to the volume of data, and I must re-emphasize the fact that neither I nor anyone else is capable of achieving the needed synthesis alone and unaided (Potter 1956). The present effort accordingly invites needed discussion.

### REFERENCES

Abramson, S., Miller, R. G. & Phillips, R. A. (1977) The identification in adult bone marrow of pluripotent and restricted stem cells of the myeloid and lymphoid systems. *J. Exp. Med.*, **145**, 1567.

Alexander, P. (1972) Foetal "antigens" in cancer. *Nature*, **235**, 137, 181.

Becker, A. J., McCulloch, E. A. & Till, J. E. (1963) Cytological demonstration of the clonal nature of spleen colonies derived from transplanted mouse marrow cells. *Nature*, **197**, 452.

Bradley, T. R. & Metcalf, D. (1966) The growth of mouse bone marrow cells *in vitro*. *Aust. J. Exp. Biol. Med. Sci.*, **44**, 287.

Brookes, J. H. & Osgood, E. E. (1959) Long-term mixed cultures of human hemic cells with granulocytic, lymphocytic, plasmacytic, and erythrocytic series represented. *Blood*, **14**, 803.

Broxmeyer, H. E., Baker, F. L. & Galbraith, P. R. (1976) *In vitro* regulation of granulopoiesis in human leukemia: application of an assay for colony inhibiting cells. *Blood*, **47**, 389.

Broxmeyer, H. E., Moore, M. A. S. & Ralph, P. (1977) Cell-free granulocyte colony inhibiting activity derived from human polymorphonuclear neutrophils. *Exp. Hematol.*, **5**, 87.

Burchenal, J. H. (1970) Obituary: David A. Karnovsky. *Cancer Res.*, **30**, 548.

Butcher, F. R. & Potter, V. R. (1972) Control of the adenosine 3′,5′-monophosphate-adenyl cyclase system in the livers of developing rats. *Cancer Res.*, **32**, 2141.

Butcher, F. R., Scott, D. F., Potter, V. R. & Morris, H. P. (1972) Endocrine control of cyclic adenosine 3′,5′-monophosphate levels in several Morris hepatomas. *Cancer Res.*, **32**, 2135.

Cohen, P. (1973) The subunit structure of rabbit-skeletal-muscle phosphorylase kinase, and the molecular basis of its activation reactions. *Eur. J. Biochem.*, **34**, 1.

Cohen, P. (1976) *Control of Enzyme Activity*. London: Chapman and Hall.

Coleman, J. R. (1976) Book review of "Cell Cycle and Cell Differentiation". Eds. J. Reinert and H. Holzer. New York: Springer-Verlag. 1975. *Science*, **191**, 844.

Crone, M. & Itzhaki, S. (1965) On the relative functioning of the pathways for formation of thymidine nucleotides in the regenerating liver and spleen of the rat. *Biochim. Biophys. Acta*, **95**, 8.

DeNechaud, B. & Uriel, J. (1971) Antigènes cellulaires transitoires du foie de rat. I. Sécrétion et synthese des protéines sériques foetospecifiques au cours du developement et de la régénération hépatiques. *Int. J. Cancer*, **8**, 71.

Dutcher, R. M. & Chieco-Bianchi, L. (1973) *Unifying Concepts of Leukemia*. Basel: S. Karger.

Fibach, E., Hayashi, M. & Sachs, L. (1973) Control of normal differentiation of myeloid leukemic cells to macrophages and granulocytes. *Proc. Natl. Acad. Sci., U.S.A.*, **70**, 343.

Gorman, R. R., Bunting, S. & Miller, O. V. (1977) Modulation of human platelet adenylate cyclase by prostacyclin (PGX). *Prostaglandins*, **13**, 377.

Greengard, P. (1978) Phosphorylated proteins as physiological effectors. *Science*, **199**, 146.

Gruber, D. F., Zucali, J. R. & Mirand, E. A. (1977) Identification of erythropoietin producing cells in fetal mouse liver cultures. *Exp. Hematol.*, **5**, 392.

HECHT, L. I. & POTTER, V. R. (1956) Nucleic acid metabolism in regenerating rat liver. III. Intermediates in the synthesis of DNA pyrimidine nucleotides. *Cancer Res.*, **16**, 999.

HIRAI, H., NISHI, S., WATABE, H. & TSUKADA, Y. (1973) Some chemical, experimental, and clinical investigations of α-fetoprotein. *Gann Monogr.*, **14**, 168.

HOLLIDAY, R. & PUGH, J. E. (1975) DNA modification mechanisms and gene activity during development. *Science*, **187**, 226.

HOWARD, A. & PELC, S. R. (1953) Synthesis of DNA in normal and irradiated cells and its relation to chromosome breakage. *Heredity*, **6**, (Suppl.), 261.

KELLY, D. S. & POTTER, V. R. (1977) Amino acid concentrating ability of slowly growing autochthonous hepatomas and host livers. *Biochem. Biophys. Res. Comm.*, **75**, 219.

KORN, A. P., HENKELMAN, R. M., OTTENSMEYER, F. P. & TILL, J. E. (1973) Investigation of a stochastic model of haemopoiesis. *Exp. Hematol.*, **1**, 362.

KROES, R., SONTAG, J. M., SELL, S., WILLIAMS, G. M. & WEISBURGER, J. H. (1975) Elevated concentrations of serum α-fetoprotein in rats with chemically induced liver tumors. *Cancer Res.*, **35**, 1214.

KURLAND, J. I., BOCKMAN, R. S., BROXMEYER, H. E. & MOORE, M. A. S. (1978) Limitation of excessive myelopoiesis by the intrinsic modulation of macrophage-derived prostaglandin E. *Science*, **199**, 552.

KURLAND, J. & MOORE, M. A. S. (1977*a*) Modulation of hemopoiesis by prostaglandins. *Exp. Hematol.*, **5**, 357.

KURLAND, J. & MOORE, M. A. S. (1977*b*) The regulatory role of the macrophage in normal and neoplastic hemopoiesis. In *Experimental Hematology Today.* Eds. Baum, S. J. and Ledney, G. D. New York: Springer-Verlag. p. 51.

LAISHES, B. A. & FARBER, E. (1978) The transfer of viable putative preneoplastic hepatocytes to the livers of syngeneic host rats. *J. Natl. Cancer Inst.* (in press).

LAISHES, B. A., ROBERTS, E. & FARBER, E. (1978) *In vitro* measurement of carcinogen-resistant liver cells during hepatocarcinogenesis. *Int. J. Cancer*, **21**, 186.

LAJTHA, L. G. (1963) On the concept of the cell cycle. *J. Cell. Comp. Physiol.*, **62**, (Suppl.), 143.

LEFFERT, H. L., KOCH, K. S. & RUBALCAVA, B. (1976) Present paradoxes in the environmental control of hepatic proliferation. *Cancer Res.*, **36**, 4250.

LOTEM, J. & SACHS, L. (1974) Different blocks in the differentiation of myeloid leukemic cells. *Proc. Natl. Acad. Sci., U.S.A.*, **71**, 3507.

MCCULLOCH, E. A., MAK, T. W., PRICE, G. B. & TILL, J. E. (1974) Organisation and communication in populations of normal and leukemic hemopoietic cells. *Biochim. Biophys. Acta*, **355**, 260.

MACMANUS, J. P. & BRACELAND, B. M. (1976) A connection between the production of prostaglandins during liver regeneration and the DNA synthetic response. *Prostaglandins*, **11**, 609.

MAEDA, S. & SACHS, L. (1978) Control of normal differentiation of myeloid leukemic cells. XIII. Inducibility for some stages of differentiation by dimethylsulfoxide and its dissociation from inducibility by MGI. *J. Cell. Physiol.*, **94**, 181.

MALEY, F. & MALEY, G. F. (1960) Nucleotide interconversions. II. Elevation of deoxycytidylic deaminase and thymidylate synthetase in regenerating rat liver. *J. Biol. Chem.*, **235**, 2968.

MALEY, F. & MALEY, G. F. (1961) The presence of deoxycytidylic deaminase in normal adult rat liver. *Biochim. Biphys. Acta*, **47**, 181.

MALEY, F. & MALEY, G. F. (1961) Nucleotide interconversions. IV. Activities of deoxycytidylic acid deaminase and thymidylate synthetase in normal rat liver and hepatomas. *Cancer Res.*, **21**, 1421.

MENDELSOHN, M. L. & DETHLEFSON, L. A. (1973) Cell kinetics of breast cancer: the turnover of nonproliferating cells. *Recent Results Cancer Res.*, **42**, 73.

METCALF, D. (1973) Regulation of granulocyte and monocyte-macrophage proliferation by colony stimulating factor (CSF): A review. *Exp. Hematol.*, **1**, 185.

MORRIS, H. P. & MERANZE, D. R. (1974) Induction and some characteristics of "minimal deviation" and other transplantable rat hepatomas. *Recent Results Cancer Res.*, **44**, 103.

OSGOOD, E. E. (1957) A unifying concept of the etiology of the leukemias, lymphomas, and cancers. *J. Natl. Cancer Inst.*, **18**, 155.

OSGOOD, E. E. (1959) Regulation of cell proliferation. In *The Kinetics of Cellular Proliferation.* Ed. Stohlman, F. New York: Grune and Stratton. p. 282.

OSGOOD, E. E. & KRIPPAEHNE, M. L. (1955) The gradient tissue culture method. *Exp. Cell Res.*, **9**, 116.

PACE-ASCIAK, C. R. (1977) Minireview: oxidative biotransformations of arachidonic acid. *Prostaglandins*, **13**, 811.

PARIZA, M. W., BUTCHER, F. R., KLETZIEN, R. F., BECKER, J. E. & POTTER, V. R. (1976) Induction and decay of glucagon-induced amino acid transport in primary cultures of adult rat liver cells: paradoxical effects of cycloheximide and puromycin. *Proc. Natl. Acad. Sci., U.S.A.*, **73**, 4511.

PIERCE, G. B., NAKANE, P. K. & MAZURKIEWICZ, J. E. (1974) Natural history of malignant stem cells. In *Differentiation and Control of Malignancy of Tumor Cells.* Eds. Nakahara, W., Ono, T., Sugimura, T. and Sugano, H. Tokyo: Univ. Tokyo Press. p. 453.

PITOT, H. C., BARSNESS, L., GOLDSWORTHY, T. & KITAGAWA, T. (1978) Biochemical characterization of stages of hepatocarcinogenesis after a single dose of diethylnitrosamine. *Nature*, **271**, 456.

PITOT, H. C. & POTTER, V. R. (1960) An enzymic study of the cellular origin of the Dunning and the Novikoff hepatomas in the rat. *Biochim. Biophys. Acta*, **40**, 537.

PLUZNIK, D. H. & SACHS, L. (1965) The cloning of normal "mast" cells in tissue culture. *J. Cell. Comp. Physiol.*, **66**, 319.

PLUZNIK, D. H. & SACHS, L. (1966) The induction of clones of normal mast cells by a substance from conditioned medium. *Exp. Cell Res.*, **43**, 553.

POTTER, V. R. (1956) Guest editorial: A plea for formal support for study and reflection. *Cancer Res.*, **16**, 725.

POTTER, V. R. (1964) Models as aids to communication. *Natl. Cancer Inst. Monogr.*, **13**, 111.

POTTER, V. R. (1968*a*) Mechanisms of carcinogenesis in relation to studies on minimal deviation hepatomas. In *Exploitable Molecular Mechanisms and Neoplasia.* Austin: Univ. of Texas Press. p. 587.

POTTER, V. R. (1968*b*) Recent trends in cancer biochemistry: the importance of studies on fetal tissue. *Can. Cancer Conf.*, **8**, 9.

POTTER, V. R. (1974) Epilogue. In *Differentiation and Control of Malignancy of Tumor Cells*. Eds. Nakahara, W., Ono, T., Sugimura, T. and Sugano, S. Tokyo: Univ. Tokyo Press. p. 553.

POTTER, V. R. & ELVEHJEM, C. A. (1936) A modified method for the study of tissue oxidations. *J. Biol. Chem.*, **114**, 495.

POTTER, V. R., WALKER, P. R. & GOODMAN, J. I. (1972) Survey of current studies on oncogeny as blocked ontogeny: isozyme changes in livers of rats fed 3′-methyl-4-dimethylaminoazobenzene with collateral studies on DNA stability. *Gann Monogr. Cancer Res.*, **13**, 121.

REYNOLDS, R. D., SCOTT, D. F., POTTER, V. R. & MORRIS, H. P. (1971) Induction of tyrosine aminotransferase and amino acid transport in Morris hepatomas and in adult and neonatal rat liver. *Cancer Res.*, **31**, 1580.

RICHARDS, W. L. & POTTER, V. R. (1978) Significance of diversity of phenotypic expression of gamma-glutamyl transpeptidase (GGT) in rat hepatomas and liver systems in relation to experiments with alpha-naphthylisothiocyanate (ANIT). *Proc. Am. Assoc. Cancer Res.*, **19**, 24.

SCOTT, D. F., BUTCHER, F. R., POTTER, V. R. & MORRIS, H. P. (1972) Naturally occurring and induced levels of amino acid transport and tyrosine aminotransferase in rats bearing Morris hepatomas. *Cancer Res.*, **32**, 2127.

SELL, S. & MORRIS, H. P. (1974) Relationship of rat $\alpha_1$-fetoprotein to growth rate and chromosome composition of Morris hepatomas. *Cancer Res.*, **34**, 1413.

SELL, S., NICHOLS, M., BECKER, F. F. & LEFFERT, H. L. (1974) Hepatocyte proliferation and $\alpha_1$-fetoprotein in pregnant, neonatal, and partially hepatectomized rats. *Cancer Res.*, **34**, 865.

SENN, J. S., McCULLOCH, E. A. & TILL, J. E. (1967) Comparison of colony-forming ability of normal and leukemic human marrow in cell culture. *Lancet*, ii, 597.

SNEIDER, T. W. & POTTER, V. R. (1969) Deoxycytidylic deaminase and related enzymes of thymidine triphosphate metabolism in hepatomas and precancerous rat livers. *Adv. Enzyme Regulat.*, **7**, 375.

SNEIDER, T. W., POTTER, V. R. & MORRIS, H. P. (1969) Enzymes of thymidine triphosphate synthesis in selected Morris hepatomas. *Cancer Res.*, **29**, 40.

STANLEY, E. R., HANSEN, G., WOODCOCK, J. & METCALF, D. (1975) Colony stimulating factor and the regulation of granulopoiesis and macrophage production. *Fed. Proc.*, **34**, 2272.

STEEL, G. G., ADAMS, K. & BARRETT, J. C. (1966) Analysis of the cell population kinetics of transplanted tumors of widely differing growth rate. *Br. J. Cancer*, **20**, 784.

STEPHENSON, J. R., AXELROD, A. A., McLEOD, D. C. & SHREVE, M. M. (1971) Induction of colonies of hemoglobin-synthesizing cells by erythropoietin *in vitro*. *Proc. Natl. Acad. Sci., U.S.A.*, **68**, 1542.

STÖCKER, E. & HEINE, W.-D. (1971) Regeneration of liver parenchyma under normal and pathological conditions. *Beitr. Pathol.*, **144**, 400.

TILL, J. E. & McCULLOCH, E. A. (1961) A direct measurement of the radiation sensitivity of normal mouse bone marrow cells. *Radiat. Res.*, **14**, 213.

TILL, J. E., McCULLOCH, E. A. & SIMINOVITCH, L. (1964) A stochastic model of stem cell proliferation, based on the growth of spleen colony-forming cells. *Proc. Natl. Acad. Sci., U.S.A.*, **51**, 29.

TSANEV, R. (1975) Cell cycle and liver function. In *Cell Cycle and Cell Differentiation*. Eds. Reinert, J. and Holtzer, H. New York: Springer-Verlag. p. 197.

TSUKADA, Y. & HIRAI, H. (1975) $\alpha$-fetoprotein and albumin synthesis during the cell cycle. *Ann. N. Y. Acad. Sci.*, **259**, 37.

WALKER, P. R. & POTTER, V. R. (1972) Isozyme studies on adult, regenerating, precancerous and developing liver in relation to findings in hepatomas. *Adv. Enzyme Regulat.*, **10**, 339.

WATABE, H., HIRAI, H. & SATOH, H. (1972) $\alpha$-fetoprotein in rats transplanted with ascites hepatoma. *Gann*, **63**, 189.